# Complementary medicine products used in pregnancy and lactation and an examination of the information sources accessed pertaining to maternal health literacy: a systematic review of qualitative studies

**DOI:** 10.1186/s12906-018-2283-9

**Published:** 2018-07-31

**Authors:** Larisa Ariadne Justine Barnes, Lesley Barclay, Kirsten McCaffery, Parisa Aslani

**Affiliations:** 1 Faculty of Pharmacy, The University of Sydney, Camperdown, NSW 2006 Australia; 2 University Centre for Rural Health, The University of Sydney, PO Box 3074, Lismore, NSW 2480 Australia; 3 Sydney School of Public Health, The University of Sydney, Edward Ford Building (A27), Camperdown, NSW 2006 Australia; 4 Sydney School of Public Health, Sydney Medical School, The University of Sydney, Rm 128B, Edward Ford Building A27, Camperdown, NSW 2006 Australia; 5 Faculty of Pharmacy, The University of Sydney, Rm N502, Pharmacy & Bank Building (A15), Science Rd, Camperdown, NSW 2006 Australia

**Keywords:** Pregnancy, Lactation, Breastfeeding, Complementary medicine products, Health literacy, Culture, Medical pluralism, Health care choices

## Abstract

**Background:**

The prevalence of complementary medicine use in pregnancy and lactation has been increasingly noted internationally. This systematic review aimed to determine the complementary medicine products (CMPs) used in pregnancy and/or lactation for the benefit of the mother, the pregnancy, child and/or the breastfeeding process. Additionally, it aimed to explore the resources women used, and to examine the role of maternal health literacy in this process.

**Methods:**

Seven databases were comprehensively searched to identify studies published in peer-reviewed journals (1995–2017). Relevant data were extracted and thematic analysis undertaken to identify key themes related to the review objectives.

**Results:**

A total of 4574 articles were identified; 28 qualitative studies met the inclusion criteria. Quantitative studies were removed for a separate, concurrent review. Herbal medicines were the main CMPs identified (*n* = 21 papers) in the qualitative studies, with a smaller number examining vitamin and mineral supplements together with herbal medicines (*n* = 3), and micronutrient supplements (*n* = 3). Shared cultural knowledge and traditions, followed by women elders and health care professionals were the information sources most accessed by women when choosing to use CMPs. Women used CMPs for perceived physical, mental-emotional, spiritual and cultural benefits for their pregnancies, their own health, the health of their unborn or breastfeeding babies, and/or the breastfeeding process. Two over-arching motives were identified: 1) to protect themselves or their babies from adverse events; 2) to facilitate the normal physiological processes of pregnancy, birth and lactation. Decisions to use CMPs were made within the context of their own cultures, reflected in the locus of control regarding decision-making in pregnancy and lactation, and in the health literacy environment. Medical pluralism was very common and women navigated through and between different health care services and systems throughout their pregnancies and breastfeeding journeys.

**Conclusions:**

Pregnant and breastfeeding women use herbal medicines and micronutrient supplements for a variety of perceived benefits to their babies’ and their own holistic health. Women access a range of CMP-related information sources with shared cultural knowledge and women elders the most frequently accessed sources, followed by HCPs. Culture influences maternal health literacy and thus women’s health care choices including CMP use.

**Electronic supplementary material:**

The online version of this article (10.1186/s12906-018-2283-9) contains supplementary material, which is available to authorized users.

## Background

Medical pluralism, or the co-existence of different medical or therapeutic systems and traditions in one local setting has been recognised in most societies around the world [1]. Studies in both low and high income countries show that women routinely seek pre and postnatal health care from both traditional and allopathic providers, even when access to care from biomedically trained midwives and doctors is available [2–6]. In some places this is due to different cultural understandings of health and illness regarding specific needs for care during the reproductive phases of a woman’s life [7, 8], but can also be to receive specific services from the different forms of care sought, or for specific pregnancy or breastfeeding related concerns [9–11]. Internationally and across economic strata, the desire for holistic care has also been associated with women’s choices to use traditional or complementary medicines in pregnancy, birth and lactation [12–16]. Holism can be seen simply as the recognition and care for both the physical body and the mind and emotions [17], or be a more multifaceted concept that incorporates the health of body, mind and spirit [18]. First Nations’ concepts of holism also encompass social and cultural connections to Land, Elders, and Nation, and views political, cultural and social determinants of health as interconnected [19–21].

The prevalence of complementary medicine use in pregnancy and lactation has been increasingly noted globally. One multinational study found that of 23 countries, rates of herbal medicine use in pregnancy were the highest in Russia (69.0%), Australia (43.8%) and Poland (49.8%) [22]. A cross-sectional survey of Hispanic women in Indianapolis USA found that 14.2 and 13.0% of women surveyed began using herbal remedies in pregnancy and breastfeeding, respectively [23]. A UK study investigating various forms of complementary and alternative medicine (CAM) used in pregnancy found that 5.1% of women surveyed used dietary supplements, 34.9% used vitamins and 5.4% used herbal medicines, and that 35% of women who used CAM also visited a trained CAM practitioner [24]. Complementary medicine use in lower income countries has also been documented. For example 12% of Kenyan women living in Nairobi, and 52.4% of Malaysian women in the Tumpat district used herbal medicine in their recent pregnancies [25, 26]. Concerns with complementary medicine use in pregnancy and lactation are frequently raised for the health of the mothers, in pregnancy due to unknown effects of complementary medicine products (CMPs) on the baby in utero. Lactation is also a concern as little is known about risks associated with CMP exposure through breastmilk [27–29].

Health literacy refers to an individual’s ability to search for, understand, and apply health information when making decisions about their health [30], and influences the health care decisions women make during pregnancy and lactation. Maternal health literacy can be defined as “the cognitive and social skills that determine the motivation and ability of women to gain access to, understand and use information in ways that promote and maintain their health and that of their children” ([31], p381). In short, the knowledge, skills and confidence a woman has will influence the health care choices she makes whilst pregnant and breastfeeding. The World Health Organisation identifies four overarching factors in health literacy: (i) the health care team and system, (ii) the condition or illness, (iii) therapy (medications, lifestyle modifications, exercise prescriptions, etc.), and (iv) patient-related factors such as prior knowledge of health and health care, literacy, numeracy and communication skills and cultural background [32]. Access to appropriate information sources, as well as the ability to appraise the information obtained in order to make safe and pertinent decisions, are also key components of health literacy [33, 34].

The objectives of this systematic review were to determine what sources of information on complementary and alternative medicine products (CMPs) have been described in the literature from a range of countries, and are used in pregnancy and lactation for the benefit of the mother, the pregnancy, child and/or the breastfeeding process. The role of maternal health literacy in these practices was also examined. This paper focuses on the results from the qualitative studies included in this systematic review. It complements a concurrent synthesis of the quantitative papers looking at the same question.

## Methods

### Protocol and registration number

Details of the protocol for this systematic review were registered on PROSPERO and can be accessed at: https://www.crd.york.ac.uk/PROSPERO/display_record.asp?ID=CRD42016052283.

### Literature search strategy and criteria

An electronic search of seven databases was undertaken: AMED Allied and Complementary Medicine (via Ovid SP), CINAHL (via Ebsco), Cochrane Database of Systematic Reviews (via Ovid SP), EMBASE (via Ovid SP), Maternity and Infant Care (via Ovid SP), Medline (via Ovid SP), and PubMed. The date range was set between 1995 and 2015 to reflect developments in the field of health literacy over this time, as well as increases in the documentation of complementary medicine use in pregnancy world-wide and in complementary medicine research [35]. A second search of the seven databases was also performed to check for subsequent publications published from 2015-Jun 2017 before completing this review. A variety of terms were used to cover the four central themes of the review: pregnancy, lactation, complementary and alternative medicine products (CMPs) and health literacy. CMPs were operationally defined as ingested herbal medicines given for specific therapeutic purposes in foods, tea, decoction, tablet, capsule or ethanolic extract forms, topical herbal preparations such as herbal washes, creams or ointments, and aromatherapy oils for inhalation, as well as dietary vitamin and mineral supplements and pre and probiotic supplements. Terms within each concept (pregnancy, lactation, complementary medicine and health literacy) were combined with OR, and results from each concept combined with AND (Additional file 1). Reference lists from relevant studies and review papers were also hand searched. An initial systematic literature search was conducted and papers’ titles were screened for inclusion or exclusion based on set criteria (Table 1). This was followed by a screening of all remaining papers’ abstracts and then full text versions of papers against the same criteria. The lead author (LAJB) screened all papers by title, abstract and full text. PA participated in the screening of titles and full text papers. Differences regarding study selection were resolved by discussion between LAJB and PA. Although the transition from non-pregnant woman through conception, pregnancy, labour, birth, and the postpartum period is a continuum experienced by each childbearing woman, these different stages are described differently within the literature. For the purposes of this systematic review, the use of complementary therapies across the childbearing continuum of pregnancy, labour and birth, and breastfeeding in the postpartum period (defined as up to 24 months) [36] have been examined.

**Table 1:** Inclusion and exclusion criteria

Inclusion criteria	
1. Use of qualitative methods for data collection including focus group discussions or in-depth interviews	
2. Focus on the use of complementary medicine products as defined operationally above	
3. Described CMP use in pregnancy and/or lactation	
4. CMPs were used by the woman for the benefit of her own health in pregnancy, the pregnancy itself, the baby and/or the breastfeeding process	
5. Information sources the woman accessed with regards to the CMPs used are reported	
6. Health literacy, or related concepts, were discussed	
Exclusion criteria	
• Pre-conceptual folic acid supplementation only	
• Trials of CMPs in pregnancy or lactation (trial would have been the information source on the CMP studied)	
• Information sources not clearly identifiable	
• Potential information sources identified by the authors, but not clearly identified by participants	
• Data not collected from pregnant and breastfeeding women themselves	
• Data only collected from health care practitioners	
• Study protocols or social marketing campaigns	
• Overview or commentary papers on CAM modalities, philosophies or practices regarding women’s health	
• Overview or commentary papers on biomedical maternity care philosophies	
• Commentary papers on CMP use or the lack of uptake of recommended nutritional supplements in pregnancy, including iron, folic acid and iodine.	
• Studies where CMPs were given directly to infants, and not the breastfeeding mothers	
• Studies focussing on CAM use to treat infertility	

### Critical appraisal of reporting quality

Each paper was assessed according to the 32 item checklist *Consolidated Criteria for Reporting Qualitative Research (COREQ)* [37]. The COREQ checklist aims to assess how comprehensively and explicitly qualitative studies are reported and covers three main domains: 1) research team and reflexivity, 2) study design and 3) analysis and findings [37]. Use of the COREQ checklist guided the assessment of the rigour and methodological coherence of the included papers and contributed to the synthesis required as part of the systematic review process.

### Data extraction

All papers included were analysed comprehensively in order to extract applicable data including: author and year, country study was performed in, number of participants, data collection and analysis methods, major factors explored, CMP type discussed, childbearing stage of relevance to the CMP use, and CMP-related information sources accessed. Following this, major and minor themes were identified and data from each study was summarised within these themes with illustrating participant quotes, where relevant.

### Thematic analysis

Findings across the studies were aggregated following the methods set out by Thomas and Harden [38] and Braun and Clarke [39]. Firstly descriptive themes were developed to describe the use of CMPs by pregnant and breastfeeding women for the benefit of their own health whilst pregnant, the pregnancy, baby or breastfeeding process, and to describe the information sources women accessed when choosing to use CMPs in pregnancy and lactation. Following this, analytical themes were developed in order to delve deeper into the concept of CMP use in pregnancy and lactation and women’s access to information sources – the reasons for CMP use, the perceived and actual benefits of CMP use and what influences women to use CMPs in pregnancy and lactation.

During the coding process, it was necessary to delineate between perceived benefits of taking CMPs for the mother, the pregnancy, the growing baby, and the breastfeeding process. For clarity these perceived benefits are divided into these different themes, but it should also be recognised that there is overlap. For example, it is of benefit to the mother’s health to avoid miscarriage, but also obviously of benefit to the pregnancy. Papers were also analysed for specific results on health literacy.

## Results

### Study selection

**Fig. 1:**
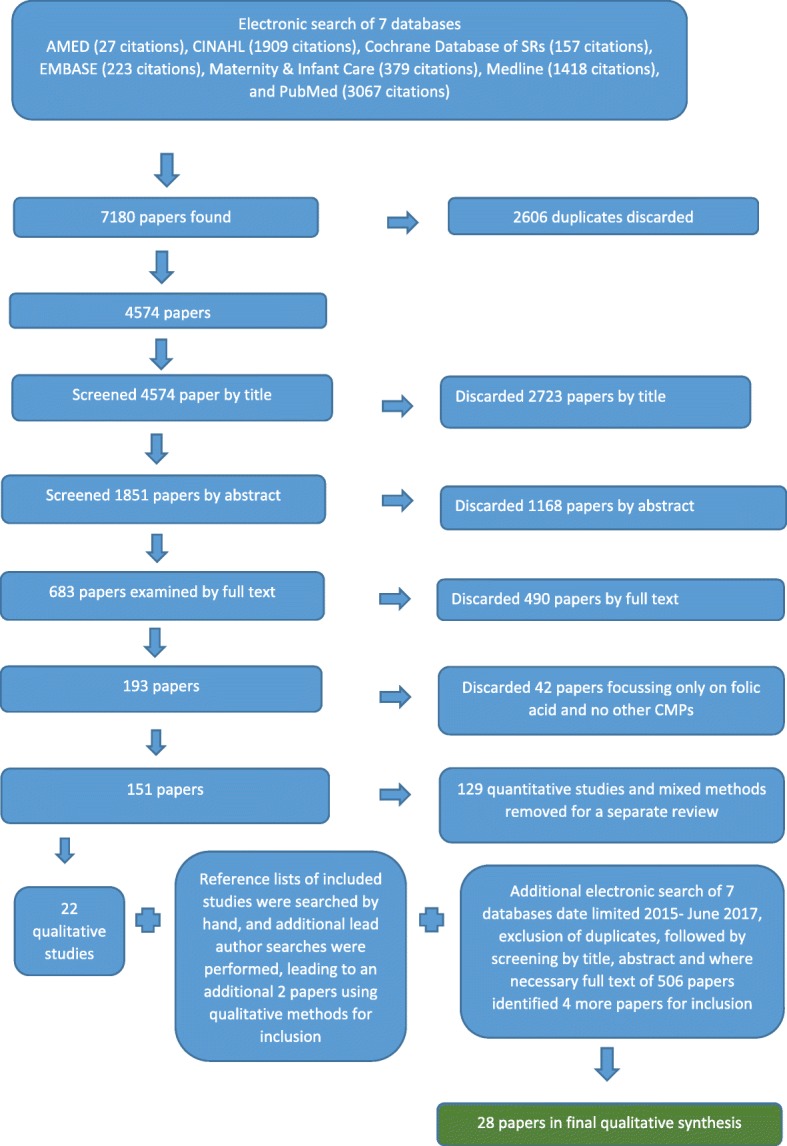
PRISMA flowchart showing review process and final number of papers in the review

The search strategy generated 4574 citations after duplicates were eliminated (Fig. 1). After reviewing titles and abstracts 683 papers were examined by full text. After studies focussing only on folic acid supplementation were removed, 22 qualitative studies were identified for inclusion. The reference lists of these 22 qualitative studies were examined by title, abstract and full text, and a further two studies were found that fulfilled the inclusion criteria. The second search of the seven databases yielded an additional 506 citations. After screening, a further four papers were identified, making a total of 28 papers covering 26 studies for inclusion in this qualitative synthesis. The three publications by Westfall [10, 40, 41] report on different aspects of one large study. Therefore, although the 28 included publications present the findings of 26 investigations, for clarity, the total number of studies will be referred to as 28 hereafter.

### COREQ appraisal results

The studies included varied in how comprehensively they fulfilled the criteria for each domain of the COREQ checklist (Additional file 2). Critical appraisal of the papers identified a number of gaps in the reporting of the papers overall.

For the first domain *Research team and reflexivity*, overall the papers reported well on who conducted the interviews and focus groups (19/28), researchers’ credentials (19/28), but less than half (13/28) reported on gender of the researchers. Interviewer characteristics, occupation, experience and whether a relationship was established between researchers and participants prior to the start of a study, and whether participants knew the researchers’ goals and reasons for doing the research were not well reported.

For domain two *Study design*, only 20/28 papers identified the methodological orientation of the research reported. Sampling method was reported clearly in 25/28 papers as was the number of participants (26/28), and to a lesser extent, place of data collection (21/28) and description of the sample (23/28). However, gaps across the studies can be seen in reporting the method of approaching participants (13/28 reported this), non-participation rates (9/28) and whether any other people were present during data collection besides researchers and participants (6/28). Data saturation was only discussed in 7/28 papers and transcripts were returned for participant comment in only 7/28 papers.

For Domain 3 *Analysis and findings,* the coding tree was only provided in 7/28 papers and in 10/28 studies participants provided feedback on the findings. Additionally, 11/28 papers reported on the number of coders. The presentation of the analysis and findings were clearly reported across most of the papers with major themes being clearly presented in the results sections of all 28 papers, and 24/28 papers also included descriptions of diverse cases or minor themes.

### Pertinent features of included studies

Table 2 describes significant features of the studies included in this synthesis.

**Table 2:** Pertinent features of included studies

Author (date)	Country (economic classification ^a^)	Number of participants	Data collection method	Data analysis method	Study aims	CMPs reported on in the papers ^b^	Stage in the continuum of childbearing reporting use of the CMP as interpreted by the author ^c^	Information sources women access for CMPs
Aborigo et al. (2012) [60]	Ghana (LMIC)	253 (including 35 women with newborn infants; 8 traditional birth attendants and local healers; 16 community leaders; 4 Focus Group Discussions [FGDs] with 8–10 grandmothers each; and 12 compound heads)	In-depth interviews [IDIs] and Focus group discussions [FGDs]	Not stated	To explore breastfeeding initiation and supplementation; cultural practices around breastfeeding initiation; and implications for the improvement of infant health	Herbal medicines	Breastfeeding	Traditional Birth Attendants (TBAs); herbalists; other local healers; women’s mothers-in-law and grandmothers; heads of households
Callister et al. (2011) [61]	Three countries: The People’s Republic of China (UPIC), Taiwain (HIC) and USA (HIC)	34 Chinese women (10 living in Guangzhou, China, 12 living Taiwan, and 12 who had immigrated to western United States.)	In-depth interviews	Not stated	Comparison of childbirth experiences of Chinese women in their countries of origin with those who had immigrated to the USA before giving birth; provide insights on Chinese women’s cultural practices and beliefs associated with giving birth for nurses and midwives in the USA.	Herbal medicines	Pregnancy and postnatal month	Shared cultural traditions; women’s mothers and mothers-in-law
Dako-Gyeke et al. (2013) [62]	Ghana (LMIC)	55 (including 17 pregnant and 15 postnatal women; 10 nurse-midwives; 2 medical doctors; 3 community members; 3 spiritualists; 1 traditional birth attendant; 1 herbalist)	In-depth interviews and Focus group discussions	Not stated	Describe the beliefs, perspectives and knowledge of pregnancy and birth of peri-Urban Ghanaian women and how these influence the health care seeking behaviour these women.	Herbal medicines	Pregnancy, labour and birth	Herbalists, TBAs and some spiritualists
Damanik (2009) [51]	Indonesia (LMIC)	64 (including 24 current mothers; 36 grandmothers)	In-depth interviews and Focus group discussions	Content analysis and Ethnography	To gather information about cultural beliefs and practices around the use of the plant Torbangun (*Coleus amboinicus Lour*) as a galactagogue by Indonesian women postnatally.	Herbal medicines	Breastfeeding and the postpartum month	Shared cultural traditions; mothers, mothers-in-law, and husbands of the new mother
Ejidokun (2000) [54]	Nigeria (LMIC)	25 (23 pregnant women; 2 health care providers who were also local grandmothers and midwives)	Focus group discussions (23 pregnant women) and in depth interviews (2 health professionals)	Thematic content analysis	Assess the knowledge, attitudes and practices related to maternal anaemia among pregnant women, health workers land the community in two Nigerian sites; to identify barriers and enablers to the use of folic acid and iron tablets by pregnant women; assess family members’ and maternal health care providers’ awareness of maternal anaemia, and how much importance they attach to it.	Iron and folic acid tablets	Pregnancy	Media: radio & printed advertisements on buses; health clinic workers; information given in places of worship like mosques.
Elter et al. (2016) [58]	Thailand (UPIC)	16 (all pregnant women)	In-depth interviews, participant observations, and a demographic record	Interpretive phenomenology	To explore first-time Thai mothers’ experiences of postpartum family practices, particularly their experiences and understandings of spiritual healing.	Herbal medicines	Early postnatal period including breastfeeding	Shared cultural knowledge; family elders
Grewal et al. (2008) [43]	Canada (HIC)	15 (postnatal women with babies less than 3 months) [N.B. 5 health care professionals and community leaders also provided recommendations based on the study findings]	In-depth interviews	Naturalistic qualitative descriptive design	Describe knowledge and cultural traditions of newly immigrated Punjabi women’s pregnancy, birth and postnatal experiences in Canada; the role of family and community in these experiences and how women incorporate these beliefs and practices into the Canadian health care system; and women’s interactions with the Canadian health care system	Herbal medicines	Labour and birth, early postnatal period and breastfeeding	Shared cultural knowledge; elders especially female family members including mothers, mothers-in-law, and sisters-in-law and husbands (if no extended family around) prepared the herbs in foods and teas for the women
Holst et al. (2009) [52]	United Kingdom (HIC)	6 pregnant women (all women were recruited from an antenatal clinic and had used herbs in pregnancy)	One Focus Group Discussion	Content analysis	To increase understanding of women’s reasons for using herbal products during pregnancy	Herbal medicines	Pregnancy	Family and friends; internet; CAM and biomedical HCPs
Juntunen et al. (2000) [7]	Tanzania (LIC)	49 (including 28 women; 21 men; informant also included a pastor; traditional healer; farmers; teachers; village health workers; traditional birth attendant; and trained hospital staff)	Open-ended interviews and participatory observation	Ethnography	To identify cultural care practices and beliefs around health protection the Bena people use throughout their lifetime	Herbal medicines	Pregnancy, labour and birth, early postnatal period	Local traditional African healers; older women in the community
Lamxay et al. (2011) [56]	Lao People’s Democratic Republic (LMIC)	30 (23 women; 7 men)	Group interviews and individual interviews	Ethnobotanical research	To study the activities and diet followed by the Kry ethnic group in Lao People’s Democratic Republic during pregnancy, childbirth and postpartum confinement period, and identify medicinal plants used during these times.	Herbal medicines	Pregnancy, labour and birth, postpartum period and breastfeeding	Husbands and other relatives, other mothers who had given birth several times and acted as assistants to the birthing woman
Liamputtong et al. (2005) [4]	Thailand (UPIC)	30 (all women - most had recently given birth; a few were currently pregnant)	In-depth interviews	Phenomenology	To understand women’s traditional beliefs and practices regarding pregnancy and childbirth among women in Northern Thailand, including the role of a traditional midwife.	Herbal medicines	Pregnancy, labour and birth	Mothers or women and men of older generations; *mor mon*, a magical healer or older man who has knowledge about magical cures and healing
Mogawane et al. (2015) [64]	South Africa (UPIC)	15 (all currently pregnant women)	Unstructured one-on-one interviews	Qualitative, explorative, descriptive, and contextual research design	Investigate the Indigenous [medical] practices of pregnant women attending the Dilokong hospital, Limpopo Province, South Africa	Herbal medicines	Pregnancy, labour and birth	Traditional African Healers, TBAs, also community elders and church leaders
Ngomane & Mulaudzi (2012) [57]	South Africa (UPIC)	12 (all currently pregnant women)	Unstructured in-depth interviews	Narrative analysis	To explore and describe the Indigenous beliefs and practices that influence late antenatal clinic attendance by pregnant women	Herbal medicines	Pregnancy, labour and birth	TBAs and family members
Obermeyer (2000) [46]	Morocco (LMIC)	151 (including 126 postnatal women; 20 modern (biomedical) health care providers; 5 traditional birth attendants)	Semi-structured in-depth interviews and observation in homes and clinics	Ethnography	Model the ethnophysiology and symbolism of pregnancy and birth in Morocco and what this implies for women’s maternal health; understand women’s health care and decision-making actions regarding birth	Herbal medicines and vitamin supplements	Pregnancy, labour and birth	Traditional midwives and traditional healers
Okafor et al. (2014) [8]	Nigeria (LMIC)	25 (all women who had delivered a baby in the previous 2 years)	Focus group discussions	No theory stated except Framework Method used to analyse data	Discover rural women’s preferred choice of health care provider for pregnancy and delivery services in Lagos, Nigeria; inform maternal health care services for rural Nigerian women	Herbal medicines	Pregnancy, labour and birth	TBAs
Rice (2000) [44]	Australia (HIC)	33 (including 27 women; three shamans; two medicine women; one magic healer)	In-depth interviews and participant observation	Ethnography	To examine cultural beliefs and practices related to the 30 day confinement period after birth in Hmong society for Hmong women now residing in Australia. Also to discuss traditional and changing patterns of childbearing for these women in their new social environment.	Herbal medicines	Breastfeeding and the postpartum month	Shared cultural knowledge; Medicine Women, Shamans, Traditional Hmong healers.
Rutakumwa & Krogman (2007) [59]	Uganda (LIC)	63 (all rural women living in Uganda)	Semi-structured interviews	Not stated, except constant comparative method of analysis to develop descriptive categories	Identify rural Ugandan women’s perspectives on their own health problems, their solutions and coping strategies, and their recommendations for improving services to suit their health needs.	Herbal medicines	Pregnancy	Shared cultural knowledge, older female family members, TBAs.
Sim et al. (2014) [55]	Australia (HIC)	20 (women all currently breastfeeding, or who had breastfed in previous 12 months; all had used herbal galactagogues)	In-depth, semi-structured interviews	Thematic analysis - transcripts were analysed using descriptive and qualitative approaches	Understand women’s perspectives and attitudes towards using herbal galactagogues during breastfeeding; understand women’s choices in using alternative medicine to promote breastfeeding; identify factors that influence their decision-making.	Herbal medicines	Breastfeeding	Internet and social-media based mothers’ groups, family and friends, trusted HCPS [biomedical HCPs, and CAM HCPs, and Lactation Consultants]
Thwala et al. (2011) [47]	Swaziland (LMIC)	15 (all women with at least 1 child, the youngest less than 2 years old)	Unstructured interviews	Ethnography	Describe the values, beliefs and childbirth practices of rural Swazi women in pregnancy, labour and the postpartum period.	Herbal medicines	Pregnancy	Shared cultural traditions, Traditional African Healers, mothers-in-law.
Waiswa et al. (2008) [45]	Uganda (LIC)	10 focus group discussions with mothers under 30 years of age, older mothers including grandmothers, fathers and childminders [but no exact number given for each FGD]; 6 key informant interviews with 6 health workers and 4 TBAs	Focus group discussions and in depth key informant interviews	Latent thematic content analysis	Assess the acceptability of Millennium Development Goals to reduce infant and maternal mortality in rural Ugandan communities; identify acceptable factors and barriers and to ante and postnatal care.	Herbal medicines	Pregnancy	Shared cultural traditions and practices; TBAs.
Warriner et al. (2014) [63]	United Kingdom (HIC)	10 (all currently pregnant women)	In-depth interviews	Not stated just thematic analysis used in analysis of transcripts	To investigate over the counter [OTC] use of complementary medicines and pharmaceutical medications in pregnancy, the role of others in influencing women’s choice to use CMPs, and how issues of choice and control influence women’s use of OTC CMPs and pharmaceuticals in pregnancy.	Vitamin and mineral supplements, homoeopathic remedies and herbal medicines available over the counter	Pregnancy	Homoeopaths, doctors and midwives, other pregnant women.
Westfall (2003a) [40] *Herbal healing*	Canada (HIC)	33 (27 currently pregnant women, of whom 26 used herbal medicines in pregnancy; 6 mentors including herbalists, authors and midwives)	In-depth interviews	Thematic analysis	To give voice to women’s self-prescription of herbal medicines in pregnancy; understand women’s perceptions of the roles and safety of herbal medicine use in pregnancy, and the choice to use herbal medicine in pregnancy.	Herbal medicines	Pregnancy	Own knowledge, own intuition, and trusted sources including books, friends, family members, biomedical HCP maternity care providers, CAM HCPs (herbalists), herbal shops, and the internet. Six mentors were listed by participants – these were midwives and childbirth educators and herbalists
Westfall (2003b) [10] *Galactagogue herbs*	Canada (HIC)	23 (women, all currently breastfeeding; 14 had used herbal galactagogues)	In-depth interviews	Thematic analysis	To discuss the potential value of five galactagogue herbs used by breastfeeding women, including the women’s own observations, historical use, safety and efficacy; inform future research.	Herbal medicines	Breastfeeding	Midwives, friends, mothers, public health nurse, doula.
Westfall (2004) [41] *Anti-emetic herbs in pregnancy*	Canada (HIC)	27 (all currently pregnant; 20 had nausea and vomiting of pregnancy, and of these 10 had used herbal medicines to treat)	In-depth interviews	Thematic analysis	Discuss the details of the herbal medicines used by women to treat pregnancy-induced nausea and vomiting.	Herbal medicines	Pregnancy	Herbalists
Wilkinson & Callister (2010) [48]	Ghana (LMIC)	24 (all pregnant women; some HCP quotes also included)	In-depth interviews and participant observation	Ethnography with the Health Belief Model	Describe the perceptions of childbirth held by Ghanaian women; inform health policy makers and health care providers to insure women receive clinically safe and culturally sensitive care.	Herbal medicines and vitamins	Pregnancy	Herbalists, biomedical midwives
Wulandari & Whelan (2011) [53]	Indonesia (Bali) (LMIC)	18 (all currently pregnant women)	In-depth interviews	Content analysis	Explore the beliefs, attitudes and behaviours of pregnant women in Bali, Indonesia	Herbal medicines and iron tablets	Pregnancy	Shared cultural knowledge, family members
Yeo et al. (2000) [49]	USA (HIC)	22 (11 couples - 11 women and their 11 husbands in were interviewed in pregnancy and then postnatally)	In-depth interviews	Ethnography	Examine Japanese couple’s perceptions and experiences of prenatal care and childbirth in Michigan, USA; explore implications for providing culturally competent care.	Pre and postnatal vitamins	Pregnancy and breastfeeding	Shared cultural knowledge, doctors, family and friends.
Young & Ali (2005) [50]	Tanzania (Zanzibar) (LIC)	52 (including 25 mothers; 27 health care workers including 4 government health officials; 3 biomedical doctors; 2 maternity ward nurses; 4 health aides; 2 pharmacists; 3 three TBAs; 1 diviner/healer; 3 traditional medicine makers; 5 employees at private pharmacies)	Informal conversations, in-depth interviews, focus group discussions and participant observation	Ethnography	Using ethnography as the basis, to describe traditional (non-biomedical) treatments for maternal iron deficiency anaemia in Zanzibar; describe women’s choices in choosing treatments; inform health planners of these choices so that and culturally appropriate care can be provided, with the aim to reduce maternal anaemia.	Traditional iron remedies and iron tablets	Pregnancy and the postpartum month	Iron tablets – hospital and nurses; Traditional remedies – traditional healers

*TBAs* Traditional Birth Assistants or Attendants, *Biomedical HCPs* biomedically trained health care practitioners - nurses, midwives, doctors and obstetricians; *CAM HCPs* Western complementary medicine health care practitioners including naturopaths and herbalists trained in Western Herbal Medicine

^a^*LIC* low income economy, *LMIC* lower middle income economy, *UPIC* upper middle income economy, *HIC* high income economy according to The World Bank Classifications [33], based on 2015 gross national income per capita

^b^ Complementary medicine type discussed in the paper, as identified by the first author (LAJB)

^c^ For the purposes of this review and analysis of the identified studies, the first author (LAJB) conceptualised the continuum of childbearing from pregnancy, birth, the early postpartum period, longer postpartum period and breastfeeding as separate but related stages

#### Geographical and economic classifications

Countries from all World Bank economic classifications [42] are represented in the sample, although the majority of studies come from countries with Low-Income Economies (LICs) or Lower-Middle-Income Economies (LMICs) classifications, and two of the studies from High-Income Economies (HICs) included actually focus on the experiences of women from poorer countries: immigrants to Canada from India, a LMIC [43]; and Hmong refugees from Thailand, an Upper-Middle-Income Economy (UPIC) living in Australia (HIC) with very low education and income levels [44]. Additionally, three of the included studies from Canada (HIC) [10, 40, 41] were from the same study, so the number of women involved from HIC backgrounds in the overall synthesis is only 143 out of more than 1075 total participants across all studies (for Waiswa et al. [45] exact numbers in the 10 FGDs were not given). Thirteen studies were from African nations: 12 from the Sub-Saharan region and one from North Africa and eight studies focussed on East or South Asian women’s experiences.

#### Theoretical frameworks for the data analysis methods

The theoretical frameworks for the studies varied: seven used an ethnographical basis [7, 44, 46–50], one combined ethnography with content analysis [51], four others used some form of content analysis [45, 52–54], and four reported using thematic analysis [10, 40, 41, 55]. Ethnobotanical research [56], narrative analysis [57], and naturalistic qualitative descriptive processes [43] provided the theoretical framework for one paper each. Phenomenology was used in two papers [4, 58]. The final five papers did not state the theoretical framework used [8, 59–62].

#### Data collection methods

Eleven studies utilised in-depth interviews only [4, 10, 40, 41, 43, 49, 53, 55, 57, 61, 63] and two studies used focus group discussions only [8, 52] to collect data. Five studies combined focus group discussions and in-depth interviews [45, 51, 54, 60, 62], four studies combined in-depth interviews with participant observation [44, 46, 48, 58], and one study combined informal conversations, in-depth interviews, focus group discussions and participant observation [50]. Data was collected using open-ended interviews and participatory observation [7], group interviews and individual interviews [56], unstructured one-on-one interviews [64], semi-structured interviews [59] and unstructured interviews [47] in the final five studies.

#### Number of participants across and within studies

The total number of participants that can be counted across all studies was 1075 but would actually be higher as exact numbers of participants were not reported in one study [45] and additional quotes from HCPs are given in another [48] without information on how many HCPs participated. Additionally, some studies [7, 45, 56, 59] included discussions with pregnant or lactating women as well as other community members, family members and health care practitioners without reporting numbers of each type of participant. Hence the total number of pregnant and/or breastfeeding women across all studies that can actually be counted is 566, but will have been larger. For those studies where the number of pregnant and lactating women is clearly stated, sample sizes ranged between six and one hundred and twenty-six, and averaged 31. Overall, there was a wide variety in the number of participants, with the smallest being a UK study with just one focus group of six women [52] and the largest including 126 semi-structured interviews with women who had recently given birth in Morocco [46]. Most studies had between 15 and 35 participants.

#### Types of CMP discussed

Herbal medicine use was the main CMP discussed, with 21/28 focussing on herbal medicines exclusively, 3/28 discussing herbal medicines and vitamin supplements [46, 48, 53], and 3/25 discussing iron and folic acid [54], pre and postnatal vitamins [49], and traditional iron remedies and iron supplements [50] respectively. In addition to vitamin and mineral supplements and herbal medicines, homoeopathic remedies were also included in one paper [63].

#### Focus on pregnancy and/or breastfeeding

**Fig. 2:**
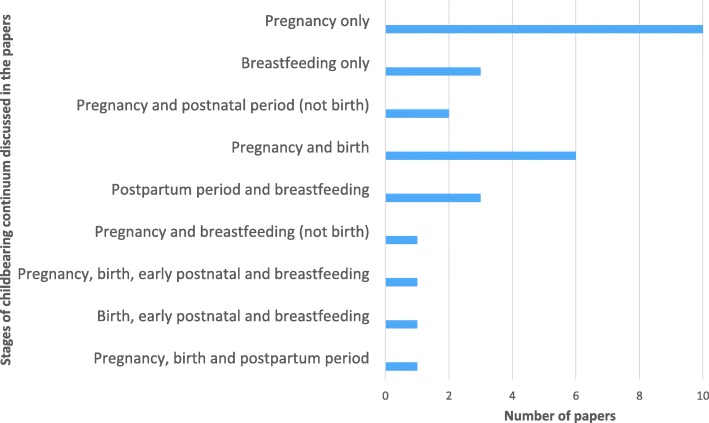
Distribution of studies focussing on CMP use during different stages of the childbearing continuum (*n* = 28) as identified by the first author (LAJB)

Although the continuum of childbearing can be conceptualised from pre-conception through pregnancy, birth, the postpartum period and breastfeeding, there was great variety in the foci of the papers included (Fig. 2). Only nine papers discussed CMP use during breastfeeding [10, 43, 44, 49, 51, 55, 56, 58, 60]. The remaining 19 papers discussed CMP use in pregnancy and other childbearing stages without reference to breastfeeding.

### Information sources accessed by women around the world

**Fig. 3:**
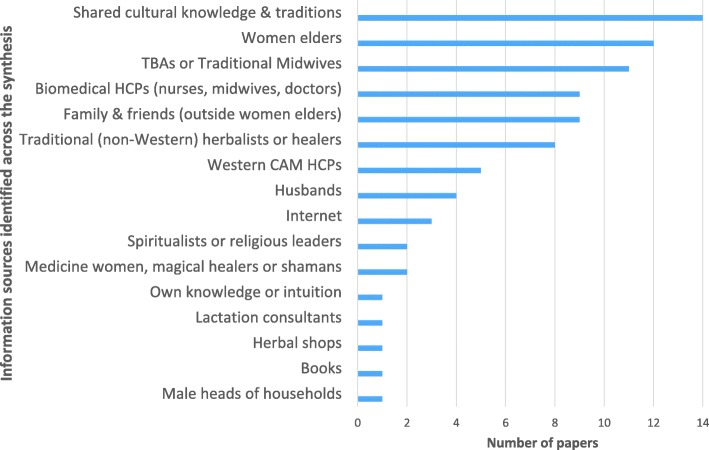
Information sources accessed by women regarding using CMPs in pregnancy and lactation across the synthesis

The information sources accessed by women when choosing to use CMPs in pregnancy and lactation are illustrated in Fig. 3 (and by country groups, see Additional file 3). Shared cultural knowledge and traditions (14 papers) followed by women elders (women’s own mothers, mothers-in-law and grandmothers, other older experienced female family members) (11 papers) were information sources identified most commonly. Following this, women accessed their health care providers for information – for women from LMIC and LIC countries and backgrounds this included Traditional Birth Assistants, traditional (non-Western) herbalists or healers, medicine women, magical healers or shamans [4, 7, 8, 44–47, 59, 60, 62, 64] but also included biomedical health care practitioners in some studies [48, 50, 54]. Similarly, women from HIC backgrounds often sought information from biomedical health care providers as well as Western herbalists or naturopathic practitioners [40, 52, 55, 63]. One significant difference between women in high income countries and low to middle income countries, was that women in HICs reported accessing CMP information via the Internet, whereas women from low and low-middle income countries did not. The studies involving immigrant women from lower income countries into HICs (Punjabi women to Canada, Hmong women to Australia) showed that the women brought their cultural traditions and knowledge with them to their new countries and that traditional knowledge and practices remained important. Similarly, Yeo, Fetters [49] found that the strong cultural beliefs held by Japanese women living in the USA influenced their willingness to take prenatal vitamin supplements. The near-universality of family and friends being reported as information sources is evident when combining the group reporting women elders and other female family members, husbands and family and friends together.

### Discussion of health literacy in the papers

For the included studies, the role of health literacy in women’s use of CMPs during pregnancy and lactation was complex. The reasons why mothers make decisions about their own and their children’s health care are influenced by women’s individual skills and abilities to access and evaluate health information, as well as individual skills and knowledge [65, 66]. None of the included studies directly measured the health literacy levels of participants and nor did any discuss findings explicitly in relation to health literacy as an over-arching concept. However, participants’ knowledge, attitudes and practices, all of which are concepts related to health literacy, were discussed.

### Knowledge, attitudes and practices

All studies discussed participants’ knowledge, attitudes and practices. ‘Health beliefs and practices’ was the most commonly discussed aspect of health (18/28 papers) followed by ‘health knowledge, attitudes and practices’ (12/28), ‘health care seeking behaviours’ (12/28) and ‘health behaviours’ (11/28). Health beliefs and practices were the greatest influence on women’s use of CMPs across the papers – women took CMPs because of perceived health benefits to themselves and/or their babies (discussed further below). The cultural importance regarding use of CMPs was also evident, especially for women from LICs and LMICs [45–47, 50, 51, 53, 54, 59, 60], but also for women in UPICs and HICs who described the importance of specific cultural practices during pregnancy, childbirth and the postpartum period [4, 43, 44, 49, 58, 61]. For many, the information regarding the cultural importance of CMP use during the childbearing continuum was passed on to them through women elders in their communities [7, 43, 44, 47, 51, 53, 57–61, 64] (also see Fig. 3).

Women’s health beliefs, practices, and health behaviours were influenced by their health knowledge and attitudes. For women in developing countries, knowledge of the biomedical model of pregnancy and birthing care was often poor. Women did not understand how regular antenatal care could help reduce their own and their babies’ risks of morbidity and mortality [45, 57, 62]. Women’s cultural knowledge regarding needs for traditional medical care along with their needs for psychosocial support led them to seek traditional care, and their albeit limited understanding of the biomedical model motivated them to access biomedical care [8, 45–48, 59, 62, 64]. In more wealthy economies, women’s engagement in medical pluralism was also discussed in relation to health beliefs and practices. Women’s perceptions of CMPs as being safer than pharmaceutical medications was explored [10, 40, 41, 52, 55, 63], as was their use of CMPs as part of efforts to increase autonomy, and self-responsibility for their own and their infants’ health [40, 55, 63]. Knowledge regarding the safety profiles of herbal medicine especially was also considered low in several of the studies across income streams [52, 53, 63], although this was usually discussed from the perspective of a biomedical outsider, with requisite concerns regarding lack of scientific testing of the CMPs being the basis of authors’ concerns.

The focus of almost half the discussions (13/28) was on how to improve patient outcomes through culturally competent care [4, 43–45, 47, 49, 50, 53, 54, 56, 57, 61, 64]. Women’s health knowledge, attitudes, beliefs, and practices were all discussed in relation to their health care seeking behaviours and especially in the poorer countries where maternal morbidity and mortality are high, in relation to their health outcomes. For these poorer communities, whole of community approaches to improving the health literacy through education and information dissemination were commonly proposed [45, 50, 53, 54, 57, 60], as often pregnant or breastfeeding women experienced significant barriers to accessing biomedical health care. These barriers included geographical isolation and/or gender inequities [47, 59, 60], as well as cultural norms that advocated family decision-making over individual decision-making and where family based care during pregnancy and the postpartum period was the norm [45, 47, 53, 58, 60]. For women in wealthier economies where culturally competent care was also discussed, the focus was more on what biomedical HCPs could do to improve provider-patient communication and understand the culturally based needs of pregnant and breastfeeding women [43, 44, 49, 61].

### Women’s use of CMPs in pregnancy and lactation and their perceived benefits

Thematic analysis revealed that women’s use of CMPs in pregnancy and lactation could be separated into several themes with associated subthemes. Additionally, women’s use of CMPs in pregnancy and lactation can be separated into two main over-arching motives, ‘Protective or preventative actions’ or ‘Facilitation of a normal process’ (Table 3). These themes and subthemes are further elaborated in Additional file 4.

**Table 3:** Thematic analysis: women’s use of CMPs in pregnancy and lactation and perceived benefits

*Use of CMPs during pregnancy*			
*Major themes*	*Subthemes*	*Over-arching motive ‘Protective or preventative action’ OR ‘Facilitation of a normal process’*	*Selected examples (full thematic analysis can be seen in* Additional file 3*)* *(in italics – participant direct quotes;* in Roman (non-italicised) - text quotes (the papers did not always include quotes)
*Women’s use of CMPs – perceived physical benefits*			
For the benefit of the pregnancy	• Prevention of vaginal bleeding and miscarriage in early pregnancy • Protect against vaginal leaking and bleeding in both early and late pregnancy	Protective or preventative action	*“At the initial stages of my pregnancy I was bleeding and I came to the hospital for drugs but it was persistent. So I went for herbal medicine and it helped me” (Focus group participant, ANC client, Madina)”* (Dako-Gyeke et al. 2013, p211) [62]
	• Ensure a safe pregnancy	Facilitation of a normal process	*“I have been advised to drink boiled herbs (Mbita) for the preservation and protection of my unborn baby, so that I may have a safe pregnancy and labour.”* (Ngomane & Mulaudzi, 2012, p34) [57]
For the benefit of the baby	• Promotion of the developing baby’s physical health - assist the baby’s intrauterine growth and support their well-being, health and vitality • Monitor the baby’s health and growth	Facilitation of a normal process	*“I think both [iron pills and herbal medicine] are important, aren’t they? I take the herbals regularly and I feel that my baby is healthy that was also what I did in my first pregnancy. I regularly took the herbals and nothing’s wrong with my baby. In fact, he was very vigorous. (Woman 6)”* (Wulandari & Whelan, 2011, p868–9) [53]
	• No perceived benefit for the use of CMPs in pregnancy – taking vitamins was incompatible with Japanese cultural beliefs around taking medications in pregnancy	Neither	*“I have been eating Japanese food in the United States just like I did in Japan when I had my first child. I never took a vitamin with my first child*. *.*. *and it did not have any bad effects on my child*. *.*. *then American doctors told me that it’s better to take vitamins*. *.*. *I don’t mind taking it, but I don’t know why I need to take it, as nothing bad happened with my first child in Japan.” (Yeo* et al.*, 2000, p194)* [49]
For the benefit of the mother	• Prevention or treatment of common illnesses associated with pregnancy like thrush and urinary tract infections • Prevention or treatment of non-pregnancy related illnesses • First line treatment of maternal danger signs in pregnancy • Protection against the development of pregnancy complications	Protective or preventative action	“The participants identified ‘*aseje’*, (a special concoction, mainly herbs) as one of the attractions of seeking care from TBAs. It is believed that the ‘*aseje’* prevents development of any complications during pregnancy and labour and keeps pregnant women healthy” (Okafor et al. 2014, p46) [8]
	• Safe support for mother’s own physical health • Treatment of maternal anaemia; provision of nourishment • Safe form of treatment for nausea and vomiting of pregnancy • Treatment of abdominal pain in pregnancy	Facilitation of a normal process	“Tonic herbs can be thought of as lying somewhere in between food and drugs; they are used therapeutically, to treat sub-clinical conditions or to prevent health degeneration. They are used to strengthen, nourish and support the body, to prevent rather than cure disease […] The most popular herb was raspberry leaf *(Rubus idaeus)* - a uterine tonic - used by 22 women.” (Westfall 2003 – herbal healing, pp26–27) [40].
For the benefit of the labour and birthing processes	• Prevention of vaginal tearing during birth and reducing risk of caesarean section • Prevention of foetal distress	Protective or preventative action	“A typical example is what is locally known as *amalagala*, a product of crushed sweet-potato leaves mixed with water. This mixture is administered to pregnant women, who bathe in it or sit on it to lessen the risk of requiring a Caesarean section or of vaginal tearing during delivery. The women did not discuss trial and error for this concoction but unanimously reported confidence in its efficacy” (Rutakumwa & Krogman, 2007) [59]
	• Use of herbal tonics to tone the uterus and strengthen it in preparation for labour • Prepare for an easy birth • Enhance or induce labour • Relieve labour pains • Induce expulsion of retained placenta • Relieve afterbirth pains	Facilitation of a normal process	“Consumption of traditional herbal medicine was also mentioned as a way of preparing for an easy birth. The traditional herbal medicine was referred to as *ya tom*. A woman must consume *ya tom* three times per day for three consecutive days. Women can purchase dried herbal medicine and boil it until it reduces to small cup quantity and drink it as tea. This is believed to make the baby strong, hence facilitating an easy birth.” (Liamputtong et al. 2005, p146) [4]
*Women’s use of CMPs in pregnancy to protect against spiritual threats to themselves and their unborn babies – perceived benefits involving spiritual protection*			
For the benefit of both mother and baby	• Protect the baby from spiritual threats that could cause physical harm including death of the foetus or preterm labour	Protective or preventative action	“All the women in this study stated that both the mother and baby might fall ill because of *kuhabula*. To prevent illness therefore, the women expressed belief in the power of traditional doctors and medicine, or divine prayer if the women or family was religious”. *.*. *[traditional medicines are taken] to make sure that the baby is protected on all fronts; protected from kuhabula [acquisition of illnesses from bad spirits in the environment] through the use of traditional medicine”* (Thwala et al., 2011, p95) [47]
*II. Use of CMPs during breastfeeding*			
*Women’s use of CMPs – perceived physical benefits*			
For the benefit of the breastfeeding process	• Increased breastmilk production – perceived and diagnosed milk insufficiency • Use of galactagogues ‘just in case’ breastmilk supply needs support • Use of galactagogues to build supply as part of a cultural tradition (note, no mention of perceived insufficiency)	Facilitation of a normal process	*“I think it’s [fenugreek] worth trying. And as for me, I certainly find that useful and reassuring that I have found something effective to increase my milk supply. As a new mum, you just never know, you never know what is coming, what problems you will encounter and I certainly did not anticipate that milk supply will be an issue. I have always thought that breastfeeding is easy and will come naturally because everyone else does it, and I wasn’t told about it being an issue”. (BW 12).* (Sim et al., 2014, p216) [55]
For the benefit of the breastfeeding process and the mother’s physical health	• Use of galactagogues supports post-birth recovery and also builds breastmilk supply	Facilitation of a normal process	“During the early postpartum period as women recovered, family members again provided certain foods that were believed to have ‘hot effects’ and bring the body into balance. These types of food are seen as essential for healing and recovery from the birthing process (arising from Ayurveda traditions), including relieving back pain, promoting menstrual flow to cleanse the body, building the mother’s milk supply, and preventing weakness and illness in later life. ‘Hot foods’ included … chai (fennel seed tea with ginger) … and other special foods … made from ‘heat-producing’ ingredients such as ginger powder, fennel seeds … and special herbs.” (Grewal, 2008, p294) [43]
		Protective or preventative action	
For the benefit of the mother’s physical health	• Expulsion of lochia through ‘uterine cleansing’ and control of postpartum bleeding • Assists in recovery after childbirth • Restoration of physical balance through heat	Facilitation of a normal process	*“You eat them [chicken herbal medicine] so that your body will settle back to normal quicker and if you don’t use them then it will take you a long time to get back to normal. The bleeding will go on for a long time and that will make you very thin. That is not good.*. *. If you bleed too long the body won’t get back to normal again and this can make you pale and skinny. If you have the chicken herbs to eat then your blood will be good and you will feel strong quickly.*. *. You eat them to give you strength and also to wash out your blood quickly too”* (Rice, 2000, p29) [44]
	• Treatment of a prolapsed uterus • Protection of the mother’s future health	Protective or preventative action	“Considered the most important Chinese cultural practice is ‘doing (or sitting) the month’ (*zuoyuezi*). … ‘Doing the month’ includes activity restrictions, avoiding ‘wind chill’ ... and eating raw ginger soup with Chinese herbs to ‘rid the body of cold’ … If such practices as described are not followed, the new mother is at risk for ‘the month disease,’ which is thought to have deleterious effects on their health for the rest of their lives (Callister et al., 2011, pp390–1) [61]
For the benefit of the breastfeeding baby	• Protection of the breastfeeding baby through the mother’s use of CMPs • Purification of mother’s breasts in preparation for breastfeeding and to ensure breastmilk is sweet	Protective or 2preventative action	“The ingestion of local herbs is used as a means of warding off any harmful effects to the baby […] To protect the baby from health problems … the newly delivered mother, her mother, and her mother-in-law - should take local drugs [herbal medicines] before the grandmother sees the baby for the first time” (Juntunen et al., 2011, p177) [7]
	• Promotion of the baby’s health through enabling the mother to continue to breastfeed	Facilitation of a normal process	“All participants seemed to have adopted the ‘breast is best’ philosophy. These women acknowledged and appreciated the health, physical and psychological benefits of breastfeeding to both mothers and infants. […] Recognition of the importance and significance of breastfeeding was identified as the main facilitator to develop perseverance and a determined attitude to breastfeed: *“I mean honestly, if drinking snake oil would make me have more breast milk I would have done it, anything that helps!”* (Sim et al., 2014, p216) [55]
*Women’s use of CMPs during breastfeeding – perceived mental-emotional benefits*			
For the benefit of the mother	• Increased self-confidence, self-empowerment and reassurance • Increases my ability for self-care	Facilitation of a normal process	“Many participants also mentioned the feeling of reassurance through the use of herbal supplements during breastfeeding, which was especially important for first-time mothers. Hence, the use of herbal galactagogue was described as a method of reassurance in the context of their own perceptions. The positive emotional impact contributed to the success of breastfeeding practices amongst the participants.” (Sim et al., 2014, p216) [55]
	• Restoration of mind-body balance	Protective or preventative action	“The herbs in hot bath, such as leaves of *Nat*, release aromatic oils, which are believed to relieve mind–heart, emotional, and psychological stress. LD said ‘*the water for a hot bath is boiled with leaves of an herb named Nat. The leaves will prevent her from feeling dizzy or being intoxicated.’* Leaves of *Nat* … can be used for treating fatigue, exhaustion, psychological and emotional imbalances, and postpartum depression [and also] to ward off a malevolent spirit and to make holy water. The women in this study used both the medicinal and supernatural properties of Nat leaves to treat the mind–heart essence” (Elter et al., 2016, p253) [58].
*Women’s use of CMPs during breastfeeding – perceived benefits involving spiritual protection*			
For the benefit of the mother	• Spiritual protection in the postpartum period	Protective or preventative action	In Thailand, *Nat* leaves are also used to ward off a malevolent spirit and to make holy water. The women in this study used both the medicinal and supernatural properties of Nat leaves to treat the mind–heart essence” (Elter et al., 2016, p253) [58]
*Women’s use of CMPs during breastfeeding – perceived cultural benefits*			
For the benefit of the mother	• Cultural cleansing rituals after childbirth	Facilitation of a normal process	“Also first-time mothers are expected to go through a cultural cleansing known as *sooru* in Kasem and *kosoto* in Nankani, regardless of the bitterness of their breastmilk. The process involves the pouring of warm herbal water over the mother for a period of 3 days if the child is a male and for 4 days if the child is female” (Aborigo et al. 2012, p76) [60]
*III. Additional themes relating to perceived benefits of women’s use of CMPs throughout the childbearing continuum*			
Perceptions of safety regarding CMP use in pregnancy and lactation	• Complementary medicines are safer than pharmaceutical medications • Receiving reassurance that herbal medicines are safe during pregnancy and breastfeeding	Protective or preventative action	*‘I am certainly not opposed to the idea of using herbs during breastfeeding, as long as I know and have checked with my child health nurses and doctors or even ringing up a pharmacist’ (BW 12)*” (Sim et al., 2014, p216) [55]
Using both CMPs and concurrently accessing biomedical care promotes best care for both mother and baby	• Better management of maternity complications in pregnancy and birth • Protection of the baby from diseases understood to arise from spiritual causes as well as from diseases treatable with biomedical medicines	Protective or preventative action	*“I use traditional medicines during the pregnancy … I also go to the hospital every month to have check-ups. They give me pills which I take home to drink together with the traditional medicines [...I use both traditional medicines and hospital medicines] to make sure that the baby is protected on all fronts; protected from kuhabula [acquisition of illnesses from bad spirits in the environment] through the use of traditional medicine as well as protected from the hospital diseases by using their modern medicine.”* (Thwala et al., 2012, p95) [69]

## Discussion

All mothers want what is best for themselves and their unborn and breastfeeding babies and this review has identified that mothers from a range of economically advantaged countries use CMPs to help facilitate this. Underpinning this desire and the decision-making associated with it are several factors: a woman’s individual health literacy, the health literacy environments she moves in, her own culture and the cultures at play in the health literacy environment, considerations of safety and where the locus of control regarding decision making in pregnancy and lactation sits.

### Culture, health literacy and holistic health

This review’s identification of shared cultural knowledge as a major information source for women choosing to use CMPs in pregnancy and lactation warrants further discussion of culture, health literacy and holistic health.

**Fig. 4:**
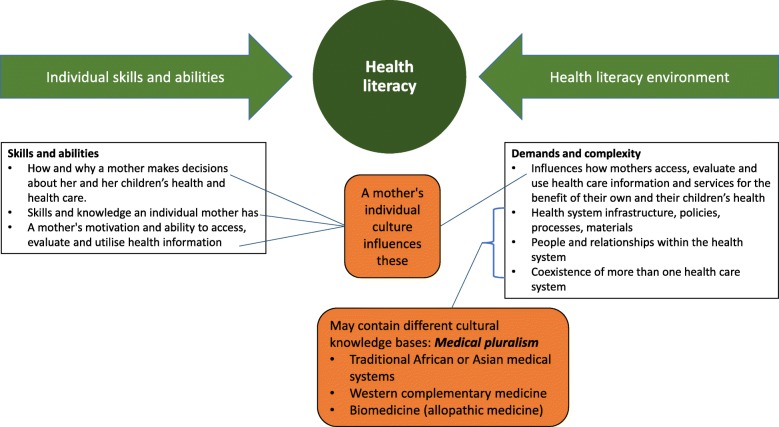
Impact of culture on health literacy, modified from Parker [66] and the Australian Commission on Safety and Quality in Health Care’s working definition of health literacy [65]

The United Nations Educational, Scientific and Cultural Organization defines culture as “the set of distinctive spiritual, material, intellectual and emotional features of society or a social group, and that it encompasses, in addition to art and literature, lifestyles, ways of living together, value systems, traditions and beliefs” [67]. This definition has been accepted by the World Health Organisation’s expert group on the cultural contexts of health and wellbeing [68]. Culture is a way of life, and can include religious, social or ethnic characteristics, but is also dynamic as values and practices can change over time. It is also important to acknowledge that all kinds of knowledge are cultural, including the practices of traditional health care systems, Western complementary health care systems, and scientific and biomedical practices [68]. A mother’s own culture influences both her individual health literacy skills and abilities, and how she accesses, evaluates and uses health care information and services in her health literacy environment when making decisions about her own and her children’s health. Additionally she may also encounter different cultural knowledge bases within both the health system infrastructure and in the people and relationships within the health literacy environment including other care-givers, the health care team and systems accessed, each with their own personal and medical cultural knowledge bases [31, 32, 65]. Thus it can be argued that women make the decisions to use CMPs in pregnancy and lactation both within the context of their own cultures and the cultures of the health literacy environment. This is illustrated in Fig. 4 which builds on Parker’s [66] model, used by the Australian Commission on Safety and Quality in Health Care in their working definition of health literacy [65]. The cultural components of health literacy and the ways they impact on individual health literacy and the health literacy environments are depicted in the orange boxes added to the original model (in green and white). In this way, the original model is expanded to include both (i) an individual mother’s culture, and how her culture influences the ways she uses her skills and abilities to access and interpret health information; and (ii) the different cultural knowledge bases extant in the health literacy environment in which she moves.

‘Protection and prevention’ and ‘Facilitation of normal physiological processes’.

Pregnant and lactating women in the countries sampled choose to use complementary medicine products based on two over-arching themes identified in this synthesis, *‘Protective or preventative action’ and/or ‘Facilitation of a normal process’.* Women’s motivation to use CMPs is based on the desire to both protect themselves and their babies from adverse events, and to facilitate the normal physiological processes of pregnancy, birth and breastfeeding. Women attempted to prevent adverse outcomes in pregnancy including miscarriage or malformation of the baby, ill health of the mother during pregnancy, and to prevent foetal distress and vaginal tearing in labour and birth. Additionally, CMPs were used to prevent future health problems for both mother and baby through the restoration of the mother’s health in the postnatal period and the establishment of breastfeeding. Whilst this synthesis predominantly identified perceived physical benefits relating to CMP use in pregnancy and lactation, perceived mental-emotional, cultural and spiritual benefits were also found. Again, the impact of culture cannot be underestimated when examining women’s health care choices in pregnancy and lactation. Whilst pregnancy, labour, birth and breastfeeding are physiologically comparable for all women, there is great variety in the social and cultural contexts within which these events occur, as well as in the individual customs, beliefs, morals and values women will bring to their individual experiences [7, 47, 69]. A woman’s cultural heritage and her cultural environment will influence her health care decisions in pregnancy and lactation [61, 70]. Finlayson & Downe’s [71] systematic review found that cultural beliefs regarding the need to protect a pregnancy from supernatural threats, combined with women’s preferences for traditional medicines, contributed to the low use of biomedical antenatal services in LICs and LMICs. Also contributing to this low utilisation was the commonly held cultural view of pregnancy as a normal physiological state, as opposed to a biomedical perception of pregnancy as a risky situation [71]. These results support the current review’s identification of the two overarching motivating themes ‘protection and prevention’ and ‘facilitation of normal physiological processes’ as strong motivators for women’s use of CMPs during pregnancy and breastfeeding for women in developing economies. Studies from LIC and LMIC countries included in the present review also identified that traditional and cultural beliefs contribute to CMP use in pregnancy and lactation, and that women view herbal and traditional medicines as being safer, more effective, affordable and more easily accessed than pharmaceutical medications, [47, 57, 62, 72]. Regarding women in HICs, motivations for their CMP use during pregnancy have been examined in four systematic reviews. Pallivalappila et al. [73] were unable to make definitive conclusions regarding pregnant women’s motivations regarding use of complementary medicine, or their perceptions of the effectiveness and safety of CMPs, due to substantial flaws in study design and reporting. However, three other reviews of CMP use by pregnant women in HICs [12–14] did find links between CMP use and women’s preferences for holistic approaches to health, along with women’s perceptions that use of complementary medicine facilitated better health, wellbeing and quality of life in pregnancy, and could help them prepare for a normal labour and birth. In line with the theme ‘facilitation of normal physiological processes’, women’s desire for autonomy and control over individual pregnancy health were also identified as motivating factors for women’s use of CMPs [12–14]. Consistent with the theme ‘protection and prevention’ Adams et al.’s [12] review also identified that women perceived their CMPs to be safer than pharmaceutical prescriptions when using CMPs to relieve pregnancy-related complaints.

#### Locus of control, culture and CMP use in pregnancy and lactation

Studies examining the health locus of control aim to describe what health beliefs influence people’s health behaviours [74]. For pregnancy this could include measuring perceived responsibility pregnant women hold (internal locus of control) and the extent external forces like chance and health professionals (termed ‘powerful others’) will affect the health outcomes of their babies [75, 76]. For pregnant and breastfeeding women from LIC, LMIC and UPIC countries, powerful others also included their mothers and mothers-in-law and other extended family members who often provided both antenatal and postpartum care within a context of culturally prescribed practices. In contrast, for women from HICs, the use of CMPs was associated with increasing autonomy and taking self-responsibility for their own, and their babies’ health [10, 55, 63]. This finding has also been documented in other qualitative and quantitative CAM research [77–79]. Locus of control can be seen as part of the wider cultural context and differs between cultures and for women living in countries of low versus high economic backgrounds.

**Fig. 5:**
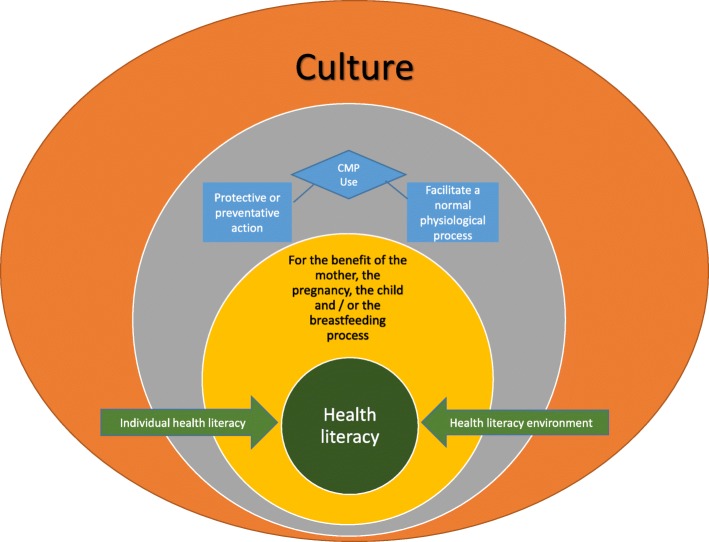
Health literacy and women’s decisions to use CMPs in pregnancy and lactation, within the overarching influence of culture, modified from Parker [66] and the Australian Commission on Safety and Quality in Health Care’s working definition of health literacy [65]

Figure 5 illustrates how the two over-arching motivators for CMP use, *‘Protective or preventative action’ or ‘Facilitation of a normal process’,* and considerations of locus of control sit within the context of culture and its influence on health literacy. Pregnant and lactating women use CMPs for their perceived benefits for the mother, the pregnancy, the child and/or the breastfeeding process. Overlaying but also integral to this is the interactive model of health literacy [65, 66] which illustrates how each individual woman is influenced by her individual health literacy and her health literacy environment. Culture is integral to both these components: it influences a woman’s individual health literacy, and different cultural influences come into play at different levels of the model, including within different elements of the health literacy environment.

### Medical pluralism and considerations of cultural influences on health care decision-making in pregnancy and lactation

The concurrent use of CAM and biomedicine has been well documented in many cultures [17, 80] and in pregnancy and lactation around the world [45, 47, 81–83]. This synthesis also highlights that many women used CMPs within a context of *medical pluralism*, defined as the co-occurrence of different medical or therapeutic systems and traditions in one local setting [1, 11]. Medical pluralism can also be seen as a patient centred model where individual consumers chose the level of integration between co-occurring health systems, which in turn facilitates a recognition of paradigm differences between biomedicine and complementary or traditional medical systems [84]. Both the autonomy of the individual woman, and the integrity of the different treatment systems she moves through are maintained [84]. Similarly to this current review, medical pluralism was identified as a contributor to women’s choices in Nagata et al.’s [80] systematic review investigating social determinants of iron supplementation in women of reproductive age. Herbal medicines and home remedies were identified as being popular, and often utilised more readily in areas where medical pluralism allowed women to choose between these and biomedical or public health measures. The success of iron supplementation often depended on collaboration or mutual respect between biomedical and traditional medical or complementary medicine systems of healing [80]. Yixi & Rancine’s [85] integrative review of the healthcare experiences of Chinese women immigrants in English-speaking countries also found that women embraced medical pluralism and utilised both Traditional Chinese and the Western biomedical healthcare approaches in a pragmatic way that allowed them to expand their healthcare choices and gave them more comprehensive ways of understanding and managing their health concerns. The current review also showed that women’s engagement with medical pluralism increased their healthcare choices in pregnancy and lactation. Additionally, it was found that women’s health literacy environments also reflect medical pluralism as the important information sources many accessed included biomedical HCPs as well as traditional healers and Western complementary medicine practitioners. As a result, for most of the studies, women navigated through and between different heath care services and systems, seeking and receiving care from both biomedical practitioners and CAM or traditional medical practitioners. However, this does not mean that women asked all their biomedical, traditional or CAM HCPs for information on CMPs. In studies involving women from East or South Asia (Indonesia, Thailand, China, Lao and India), HCPs were not identified as information sources on CMPs, rather shared cultural knowledge, women elders or other family members were the important information sources on herbal medicines [4, 43, 44, 51, 53, 56, 58, 61]. However, except for the study on Kry women from Lao [56], the women in all these studies did engage in medical pluralism as it is evident from the papers that they accessed biomedical care whilst pregnant or breastfeeding. Additionally, their exclusion of HCPs as information sources for CMPs may reflect more the questions asked to investigate the aims of each study, as other studies included in this synthesis showed that biomedical and traditional health care practitioners were important sources of information on CMPs for pregnant and lactating women originating from East Asia (Lao and Japan) [44, 49].

#### Medical pluralism and the concept of holism

It is useful to examine the reasons behind women’s engagement in medical pluralism further. MacArtney and Wahlberg [17] argue that some opponents of complementary medicine frame CAM users as ignorant of scientific methods of research, deceived by false advertising or claims, or irrational believers with a distrust of science itself, and view CAM practitioners or advocates as immoral in offering placebo or inferior treatments in place of biomedical options. This judgemental approach is unhelpful as it prevents any understanding of the evidence for why people choose to use CAM or CMPs, and polarises the debate into automatically generated positive or negative responses [17]. It also discounts the evidence showing that engagement in medical pluralism is common, and disregards wider issues that may be at play including individual health literacy and the health literacy environment, factors that play important roles in the wellbeing of women and their babies. As discussed above, engagement with plural medical systems can be seen to expand a woman’s healthcare choices [80, 85] and to determine the information sources she accesses as part of her health literacy environment when seeking to support her own and her children’s health. Conversely, there are also risks women may miss out on important health-promoting care if co-occurring medical systems are perceived to be dichotomous or in conflict [54, 80] Women’s engagement with holistic health is also a factor that plays an important role in the wellbeing of women and their babies.

There are diverse interpretations regarding the concept of holism. The simplest concept of holism, common in both CAM and biomedical texts, recognises both the physical body and the mind and emotions, originally separated in Descartes’ philosophy [17]. Some CAM texts build on this to include connections between the body, mind and spirit and/or discussions of wider social and political contexts of health [18]. First Nations’ concepts of holistic health encompass an individual’s physical, mental-emotional, spiritual, social and cultural connections to health (including connections to Land, Elders, and Nation), and see political, cultural and social determinants of health as interconnected [19–21]. The importance of information from women elders, from family and friends, and other lay people can be put into the context of the broad definitions of holistic health such as defined by First Nations’ concepts. The concept of locus of control may play a factor in women’s health care decisions, the accessing of a variety of information sources, including interpersonal, non-health care professional relations and cultural information, can likewise be viewed within these broader concepts of holism whilst also considering medical pluralism. A mother may seek help for a health care concern from several sources depending on which recommendations she feels will be safe and effective, what sources she can access, and which sources support her worldview and understandings of health and illness. If seeking support from a clinician, rapport and trust may also play a role [86]. Empowerment may also play a role, especially for women from HICs [10, 55, 77, 86].

### Safety considerations in women’s choices to use CMPs in pregnancy and lactation

This synthesis revealed that for women from HICs, the use of CMPs throughout the childbearing continuum was associated with the perceptions that CMPs were safer than pharmaceutical medications, and that these women sought reassurance that the herbal medicines they used were safe for pregnancy and breastfeeding. This perception of safety has been found previously in systematic reviews on the use of CAM generally in HIC populations [87, 88] but may not have been explored adequately in developing countries. Although the perception of safety was not a large theme explaining women’s use of CMPs in developing countries, this synthesis did reveal that for women from some LMICs and UPICs, safety for mother and baby was believed to be facilitated by the concurrent use of biomedical care with CMPs, including the prevention of maternity complications in pregnancy and birth, and protection from diseases arising from spiritual forces not treatable with biomedical drugs.

Perceptions of safety may not always be correct. Cultural influences on health care choices cannot always be considered the safest or best outcome options. Some traditional practices may endanger the lives of both mothers and their unborn babies, especially for women in some of the studies from developing nations. As an example, for some countries, one of the perceived benefits of herbal medicine use post-birth was to expel lochia and induce ‘uterine cleansing’ [43, 44, 51]. However, inducing bleeding post-birth can be very dangerous for post-partum women, and the possibility of dying during childbirth is a reality for many of the women in the LICs and LMICs in this review [45, 48].

### Implications for policy and practice

For the large proportion of studies from economically disadvantaged countries included in this review, policy and practice implications mainly centre on reducing high maternal and infant morbidity and mortality rates. Specific national policies [46, 57], the United Nations Millennium Development Goals [89], or the World Health Organisation’s policies aimed at increasing maternal and neonatal health through education, health checks and micronutrient supplements as part of regular antenatal care [45, 50, 53, 54, 57, 62, 64] or the initiation of exclusive breastfeeding immediately post-birth [60] are discussed. Several factors are implicated in implementing policies in developing countries, including the need to identify and reduce cultural, social, geographical, economic and gender barriers to adequate biomedical antenatal, birthing and postnatal care [45–47, 59, 71, 90]. Additionally, intervention strategies aimed at promoting heath seeking behaviours to support reductions in child and maternal mortality must consider pregnant and breastfeeding women’s use of CMPs, within the context of their perceptions of health risks, the safe promotion of health and where to go for support with these [90]. This is true for women in developing nations, as well as for women in HICs. Whilst the women participants and their babies in the included studies from USA, Canada, and the UK (all HICs) were at far less risk of pregnancy and birth-related morbidity and mortality than the participants from LIC, LMIC and UPIC countries, the contexts within which these HIC women chose to use CMPs include their own cultural perceptions of how to best support their pregnancies, postnatal health and the health of their babies.

Central to discussions on policy with regard to medical pluralism is an awareness that cultural awareness, including the provision of culturally appropriate care, and consultation and involvement of the whole community is necessary if any policy changes aimed at reducing maternal and infant mortality and increasing maternal and infant survival and health are to succeed [8, 45, 48, 60, 64, 71]. Community-wide consultation is essential to successfully remove barriers to maternal and infant care [45, 57, 62, 64, 71], to identify possible solutions, and educate women’s family-members, including husbands and/or mothers-in-law, who often hold the power to make decisions regarding the health care a woman is able to seek for herself or her baby [45, 59, 60]. Policies that encourage collaboration between biomedical HCPs and TBAs, traditional herbalists and other traditional healers may help improve maternal and child health outcomes [47, 57, 59, 60]. These include policies to enable further training of Traditional Birth Attendants [8, 57, 64] in recognition that TBAs are usually the providers of culturally sensitive, affordable care, are identified as being active participants in helping promote maternal and infant health, and are ideally placed to refer and accompany women at risk to biomedical care in a timely manner when necessary [8, 47, 57, 60]. Additionally, for rural women, TBAs may often be the only HCPs they regularly see [15, 47, 48, 59]. Training of TBAs in minor surgical procedures like suturing, especially in areas where birthing often takes place outside biomedical health care institutions [59] is recommended, but policy changes to encourage more broader collaboration with TBAs and traditional healers and community health workers in education around the importance of antenatal and postnatal care, micronutrient supplementation and other important measures to improve maternal and infant health outcomes is also necessary [47, 54, 57, 59, 60].

For more economically advantaged nations, policy discussions centre on educational needs of biomedical HCPs regarding complementary medicine and CMPs due to policy and practice shifts that emphasise consumers’ rights, choices, and active involvement in health care, and providers’ responsibilities when pregnant or breastfeeding women autonomously choose to incorporate CMPs as part of their health care practices [12, 63]. In HICs policy implications call for biomedical HCPs to be better educated about CMPs, in order to be more able and willing to discuss their use openly and non-judgementally with pregnant or breastfeeding women [3, 23, 24, 52, 55, 63, 91, 92] and to realise the importance of holism to mothers as well as women’s desire for autonomy and control [55, 93, 94]. Regarding breastfeeding specifically, policy implications need to ensure HCPs receive sufficient education in lactation and in helping women to breastfeed successfully in order to be able identify and provide help with breastfeeding difficulties and improve services for breastfeeding women [55, 95–97].

An important practice implication for biomedical HCPs across all economic strata centres on balancing the provision of evidence-based biomedical care that aims to ensure the safety and health of both mother and baby, and the need to accommodate culturally different health care practices and women’s choices regarding maternal health care. The recognition of the need for culturally appropriate services is linked to the perception that provision of culturally sensitive care has the potential to enhance women’s wellbeing, and in turn the health of their babies and whole communities, provided that other social determinants of health like gender, age, income and personal and ethnic history are also taken into consideration [98–100]. Cultural understandings shape how women and their caregivers receive information and how they make health care choices [70]. The provision of culturally sensitive care facilitates communication between biomedical and other HCPs, the women they provide care for, and within the whole community. By considering and respecting women’s values and beliefs around pregnancy and childbirth, women’s use of CMPs can be discussed openly. Studies across all economic strata have shown that if a woman perceives her biomedical HCP to have negative or uninterested views on CMPs, she will not candidly discuss her use of them with her HCP [24, 48, 52, 53, 63]. For developing economies, recommendations to provide training of biomedical nurses and midwives in culturally appropriate care aim at reducing cultural barriers to biomedical care and strengthening relationships between biomedical HCPs and the whole community, including traditional practitioners [7, 47, 57]. As mutual respect and communication are facilitated through the provision of culturally appropriate care, it can also help find ways to intervene appropriately if unsafe practices are identified in pregnancy or the postnatal period [47, 58, 60].

Discussions on culturally appropriate care in HICs and UPICs again centre around promoting maternal and infant health, although the focus is less on reducing morbidity and mortality, and more on increasing biomedical HCPs’ understanding and respect for cultural differences when working with women from diverse backgrounds who engage in traditional practices that may not be taught in biomedical education [43, 44, 58, 61]. The need for cultural safety to be taught in universities and health services so that HCPs can develop a critical understanding of social determinants on both their own and others’ health is crucial if culturally sensitive care is to be provided effectively [98, 100]. Recommendations for practice include recognition of the involvement of extended family as influential care-givers in providing pregnancy and postpartum care for many women [43, 58, 61] and recognition of beliefs, experiences and practices that women engage in to promote their holistic health and recovery from childbirth, and prevent future ill-health, especially in the postnatal period [44, 51, 58, 61]. HCPs can help facilitate positive health behaviours through hands-on educational activities, by identifying and working with medical and other influential leaders in the community and women’s partners [49]. Recognising language differences as a potential barrier is also an important aspect of providing culturally competent, good quality maternity care [43, 49, 61]. The use of clear pictorial-based information when communicating with pregnant or breastfeeding women is recommended regardless of a woman’s literacy or education levels, as is the provision of health information in a woman’s own language and the use of translation services [43, 49, 61].

Future research on the inter-professional relationships between biomedical, traditional and/or CAM HCPs providing care to pregnant and breastfeeding women, as well as power dynamics between women and their non-HCP information providers, would be useful in pointing out how to make women’s reproductive journeys whilst engaging in medical pluralism safe and supportive. Additionally, future research needs to encompass explicit measurements of health literacy, and investigation of how different literacy levels impact on women’s understandings and use of CMPs in pregnancy and lactation.

## Limitations

The exclusion of articles in languages other than English is a limiting factor, as important studies from all over the world discussing CMP use in pregnancy and lactation may be missing from this review. Additionally, the proportion of studies from LIC and LMIC countries was substantially larger than those from UPIC and HIC countries. This may have increased the importance of cultural knowledge and women elders as information sources identified in this synthesis.

The hand searches identified additional papers which were not identified in the original search, possibly because of the use of different keywords encompassing the terms ‘ethnobotanical’ or ‘ethnopharmacology’. The heterogeneity in research design and methodology of the included papers in this review may also restrict the ability to make larger conclusions about CMP use in pregnancy and lactation. However, this review does provide the first qualitative synthesis regarding CMP use in pregnancy and lactation, the perceived benefits of these, and the information sources women access regarding the use of CMPs in pregnancy and lactation. This provides important information for health care planners and practitioners across the world, and emphasises the importance of culture and health literacy when working with pregnant and lactating women.

## Conclusions

This review shows that women use CMPs in pregnancy and lactation in order to optimise their own holistic health, and the health of their babies, according to the benefits they perceive to be associated with the CMPs. Herbal medicines were the most commonly reported type of CMP used, followed by vitamin and mineral supplements. Women utilise a range of information sources, with shared cultural knowledge and traditions, and women elders being the most commonly identified information sources, followed by health care practitioners. This review found that culture plays a pivotal role in women’s decisions to use CMPs. The role of maternal health literacy in explaining women’s choices to use CMPs in pregnancy and lactation is explored using an interactive health literacy framework. Women choosing to use CMPs as well as accessing biomedical care can be seen to be either supplementing biomedical care, or choosing to complement it with their CMP use for the benefit of themselves and their babies, with the aim to prevent or protect themselves and their babies from adverse events, or to facilitate the normal physiological processes of pregnancy and lactation. The influence of culture on maternal health literacy and health care choices shows that women act on beliefs and practices important to their own cultural understandings of health and illness. Biomedical maternity care providers and complementary medicine health care professionals can use this information to inform their best practice and care when working with pregnant and breastfeeding women, and to understand how and why women may make decisions to use CMPs during pregnancy and lactation.

## Additional files

Additional file 1: Example search strategy. (DOCX 16 kb)

Additional file 2: Summary of COREQ analysis of the included papers. (DOCX 25 kb)

Additional file 3: Information sources accessed by women using CMPs in pregnancy and lactation by country groupings. (DOCX 105 kb)

Additional file 4: Full thematic analysis - perceived benefits of CMP use in different stages of the childbearing continuum. (DOCX 54 kb)

## Abbreviations

Biomedical HCPs: Biomedical health care practitioners. (Biomedically trained health care practitioners - nurses, midwives, doctors and obstetricians)
CAM HCPs: Complementary medicine health care practitioners. (Western complementary medicine health care practitioners including naturopaths and herbalists trained in Western Herbal Medicine)
CAM: Complementary and alternative medicine
CMP: Complementary medicine product
CMPs: Complementary medicine products
COREQ: Consolidated Criteria for Reporting Qualitative Research
FGDs: Focus group discussions
HCPs: Health Care Professionals
HIC: High Income Economy*
HICs: High Income Economies*
IDIs: In-depth interviews
LIC: Low Income Economy*
LICs: Low Income Economies*
LMIC: Lower Middle Income Economy*
LMICs: Lower Middle Income Economies*
Non-HCP information providers: Non-health care professional information providers (including women elders, family and friends)
TBAs: Traditional Birth Assistants or Attendants
UK: United Kingdom
UPIC: Upper Middle Income Economy*
UPICs: Upper Middle Income Economies*
USA: United States of America
ᅟ: * According to The World Bank Classifications [42], based on 2015 gross national income per capita
