# Supplementary material for: Complementary medicine products used in pregnancy and lactation and an examination of the information sources accessed pertaining to maternal health literacy: a systematic review of qualitative studies
Source: BMC Complement Altern Med. 2018 Jul 31;18:229. doi: 10.1186/s12906-018-2283-9 (PMC6069845; doi:10.1186/s12906-018-2283-9)
Supplement: Supplementary file 1 — Example search strategy. (DOCX 16 kb) [file 12906_2018_2283_MOESM1_ESM.docx]

# Appendix 1: Example search strategy

***Objective: To determine what complementary and alternative medicine products are used in pregnancy and lactation for the benefit of the mother, the pregnancy, child and/or the breastfeeding process; and the information resources used regarding CMs.***

**Search for literature - Medline**

*Note: After searches were complete, studies using qualitative methods were examined separately from those using quantitative methods for the purposes of reporting.*

| ***Concept 1 (pregnancy)*** | ***Concept 2(lactation)*** |  | ***Concept 3 (CAM)*** | | | | |  | **Concept 4 (health literacy** | **Limits** |
| --- | --- | --- | --- | --- | --- | --- | --- | --- | --- | --- |
| Pregnancy/ or Pregnanc*.mp  OR pregnant.mp. or Pregnant Women/ or Pregnancy Complications/ | OR Lactation  [Included post partum period which is used for puerperium] | AND | complementary medicine*  [mapped to comp therapies as below] | OR | Herbal medicine | OR | Nutritional supplements (keyword)  OR Dietary supplements  [includes pre and probiotics, synbiotics, dried years) | AND | health education/  or consumer health information/  or health literacy/  or patient education as topic/  or prenatal education/ | Female |
| OR gestat* | OR breastfeeding  OR breast feeding  [includes breast milk expression] |  | OR Complementary therapies [comp ther, anthroposophy, holistic health, medicine traditional, naturopathy, phytotherapy] |  | OR herbal medicines (keyword search) |  | OR Dietetics |  | OR Health Promotion | Woman |
| OR parturient.mp  OR parturienc* | OR lactat* |  | OR Complementary health (keyword) |  | OR herbal therapy |  | OR Natural health product* |  | OR "attitude of health personnel"/  or attitude to health/  or health knowledge, attitudes, practice/ | Women |
| OR parturition.mp or parturition/  OR childbirth.mp  OR child birth.mp  OR child bearing.mp  OR child-bearing.mp | OR Breast |  | or alternative therap* (keyword) |  | OR Plant Preparations [is within the biological preparations category with tea] – includes plant extracts, plant oils and tea |  | OR Dietary Supplements/ or Micronutrients/ or Micronutrient*.mp. or Vitamins/ or Diet/ |  | OR Health Behavior/ or Health Behaviour.mp.  or Socioeconomic Factors/ | Humans |
| OR gravid* | OR Maternal Nutritional Physiological Phenomena |  | Aromatherapy |  | OR Herb  OR plants, medicincal  OR herb-drug interactions |  | nutritional requirements/ or recommended dietary allowances/ or nutritional status/ |  | OR Nutritional Sciences/df, ed, ph, st [Deficiency, Education, Physiology, Standards] | Adolescent |
| Prenatal Care/ or Prenatal.mp. or Prenatal Education/ or Prenatal Nutritional Physiological Phenomena/ | OR Milk, Human |  | OR Alternative medicine* (keyword search) |  | OR Botanic (keyword search) |  | OR *Nutrition Policy |  | Nutritional Sciences.mp. | Teenager |
| OR pre natal  OR pre-natal |  |  | OR Alternative health (keyword search) |  | OR beverages or tea |  | OR Mineral |  | OR self care/ or self administration/ or self medication/ | Adult |
| OR antenatal or ante-natal or ante natal (all mp) | OR Lactation Disorders  OR puerperal disorders [includes PND, lacation disorders, mastitis, etc]. |  | OR natural medic* (keyword search) |  | OR Phytotherapy |  | OR multivitamin (keyword search) |  | self efficacy.mp. or Self Efficacy/ | Age limits (13-45 years old) |
| OR periconceptual.mp  OR periconceptional.mp  OR periconceptional period.mp | OR Maternal Behavior / ph [physiology] /de [drug effects] / px [psychology] |  | OR natural remedies (keyword search) |  |  |  | OR multi-vitamin (keyword search) |  | OR self care.mp. | Infant, Newborn |
| OR pre-pregnancy.mp  OR pre pregnancy.mp  OR prepregnancy.mp | OR puerperium |  | OR wholistic health |  |  |  | OR Supplement (keyword search) |  | OR Self management OR self-management (keyword) | Pediatrics |
| OR Parity/ or parity.mp | OR Postnatal Care |  | OR Integrative Medicine |  |  |  | Recommended Daily Intake.mp. or Recommended Dietary Allowances/ |  |  | Child |
| Mothers/ or Expectant mother*.mp. | OR Prolactin |  | OR evidence based medicine  OR Evidence-based (maps to the hyphenated one) |  |  |  | Fatty Acids, Omega-3.mp. or Fatty Acids, Omega-3/ |  | OR information dissemination/  or information literacy/  or health literacy/  or information seeking behavior/ | Children |
| Labor, Obstetric/ or Obstetric Nursing/ or Delivery, Obstetric/ or Obstetric*.mp. | OR Oxytocin |  | OR traditional medical system (keyword search) |  |  |  |  |  | OR nursing research/ or patient care planning/ or patient-centered care/  OR patient centered care.mp.  OR patient centered care.mp. (keyword) |  |
| Reproduction/ or Reproduc*.mp. | OR Womens health [no apostrophe] |  | OR Nonprescription Drugs |  |  |  |  |  | OR Communication/ or communication.mp. |  |
|  | OR Feeding Methods or bottle feeding |  | OR over the counter drugs |  |  |  |  |  | OR Health Services Research |  |
|  | OR Maternal-Child Nursing OR maternal health services |  | OR over-the-counter drugs |  |  |  |  |  | OR Intention/  OR Intention.mp. |  |
|  | OR Midwifery  OR obstetric nursing [includes after birth care] |  |  |  |  |  |  |  | OR Educational Status |  |
|  | OR Maternity |  |  |  |  |  |  |  | OR Health Status |  |
|  | OR Maternal Welfare |  |  |  |  |  |  |  | OR Health Services/utilization |  |
|  | OR Mothers |  |  |  |  |  |  |  | OR Literacy {maps to Health Literacy/ or Literacy.mp. or Information Literacy/} |  |
|  | OR lactagogues  OR nutritional physiological phenomena |  |  |  |  |  |  |  | OR Numeracy (keyword) |  |
|  | OR galact**a**gogue$ |  |  |  |  |  |  |  | OR Rapid Estimate of Adult Literacy in Medicine, REALM  (keyword search) |  |
|  | OR galactagogue$ |  |  |  |  |  |  |  | OR Test of Functional Health, Literacy in Adults, TOFHLA  (keyword search) |  |
|  |  |  |  |  |  |  |  |  | OR Short form TOFHLA, S-TOFHLA  (OR Short form TOFHLA, |  |
|  |  |  |  |  |  |  |  |  | OR Schwartz–Woloshin Numeracy Test – |  |
|  |  |  |  |  |  |  |  |  | Wide Range Achievement Test, WRAT (keyword search) |  |
