# Supplementary material for: Complementary medicine products used in pregnancy and lactation and an examination of the information sources accessed pertaining to maternal health literacy: a systematic review of qualitative studies
Source: BMC Complement Altern Med. 2018 Jul 31;18:229. doi: 10.1186/s12906-018-2283-9 (PMC6069845; doi:10.1186/s12906-018-2283-9)
Supplement: Supplementary file 2 — Summary of COREQ analysis of the included papers. (DOCX 25 kb) [file 12906_2018_2283_MOESM2_ESM.docx]

# Summary of COREQ analysis of the included papers

|  | Aborigo et al. [47] | Callister et al. [48] | Dako-Gyeke et al. [49] | Damanik [39] | Ejidokun [40] | Elter et al. [45] | Grewal et al. [31] | Holst et al. [51] | Juntunen et al. [5] | Lamxay et al. [43] | Liamputtong et al. [4] | Mogawane et al. [52] | Ngomane & Mulaudzi [44] | Obermeyer [34] | Okafor et al. [6] | Rice [32] | Rutakumwa & Krogman [46] | Sim et al. [42] | Thwala et al. [35] | Waiswa et al. [33] | Warriner et al. [50] | Westfall (2003) [28] | Westfall (2003b) [8] | Westfall (2004) [29] | Wilkinson & Callister [36] | Wulandari & Whelan [41] | Yeo et al. [37] | Young & Ali [38] | | **Number Yes [Y] [total n=28]** |
| --- | --- | --- | --- | --- | --- | --- | --- | --- | --- | --- | --- | --- | --- | --- | --- | --- | --- | --- | --- | --- | --- | --- | --- | --- | --- | --- | --- | --- | --- | --- |
| **Domain 1: Research team and reflexivity - *Research team characteristics*** | | | | | | | | | | | | | | | | | | | | | | | | | | | | | | |
| 1. Interviewer/ facilitator identified | - | - | Y | - | Y | Y | Y | - | Y | Y | Y | - | - | Y | - | Y | Y | Y | Y | Y | - | Y | Y | Y | Y | Y | - | Y | **19** | |
| 1. Researcher/s’ credentials identified | Y | Y | - | Y | Y | Y | Y | - | Y | - | Y | - | Y | - | - | Y | Y | Y | Y | - | Y | Y | Y | - | - | Y | Y | Y | **19** | |
| 1. Researchers occupation at time of study | - | - | - | - | - | - | - | - | - | - | Y | - | Y | Y | - | Y | Y | Y | Y | - | - | Y | - | - | - | - | Y | - | **9** | |
| 1. Gender of the researchers | Y | Y | Y | - | Y | Y | Y | - | Y | - | Y | - | Y | - | - | - | Y | Y | - | - | - | Y | Y | Y | - | Y | - | Y | **16** | |
| 1. Experience and training of the researchers | Y | Y | - | - | - | - | Y | Y | - | - | - | - | - | - | - | - | Y | - | - | Y | - | Y | Y | - | - | - | - | - | **8** | |
| ***Relationship with participants*** | | | | | | | | | | | | | | | | | | | | | | | | | | | | | | |
| 1. Relationship established prior to study commencement? | - | - | - | - | - | - | - | Y | - | - | Y | - | Y | - | - | Y | - | - | - | - | - | - | Y | - | Y | - | - | - | **6** | |
| 1. Participant knowledge of the interviewer | - | - | - | - | - | - | - | - | - | - | - | - | - | - | - | Y | - | - | - | - | - | - | - | - | - | - | - | Y | **2** | |
| 1. Interviewer characteristics | - | - | - | - | - | - | Y | - | Y | - | - | Y | Y | Y | Y | Y | Y | - | - | - | - | - | Y | - | - | - | Y | Y | **11** | |
| **Domain 2: study design - *Theoretical framework*** | | | | | | | | | | | | | | | | | | | | | | | | | | | | | | |
| 1. Methodological orientation and theory reported | - | Y | - | Y | Y | Y | Y | Y | Y | - | Y | Y | Y | Y | - | Y | - | - | Y | Y | - | Y | Y | - | Y | Y | Y | Y | **20** | |
| ***Participant selection*** | | | | | | | | | | | | | | | | | | | | | | | | | | | | | | |
| 1. Sampling method identified | Y | Y | Y | Y | Y | Y | Y | Y | Y | Y | Y | Y | Y | - | Y | Y | Y | Y | Y | Y | Y | Y | Y | Y | Y | Y | - | - | **25** | |
| 1. Method of approach | Y | - | Y | - | - | Y | - | - | - | Y | - | - | - | - | Y | Y | Y | Y | - | - | Y | Y | Y | Y | - | - | Y | - | **13** | |
| 1. Sample size reported | Y | Y | Y | - | Y | Y | Y | Y | Y | Y | Y | Y | Y | Y | Y | Y | Y | Y | Y | - | Y | Y | Y | Y | Y | Y | Y | Y | **26** | |
| 1. Non-participation rates and reasons given | - | Y | - | - | - | Y | - | Y | - | - | Y | - | - | - | Y | - | Y | - | - | - | - | - | Y | Y | - | - | Y | - | **9** | |
| ***Setting*** | | | | | | | | | | | | | | | | | | | | | | | | | | | | | | |
| 1. Setting of data collection described | Y | Y | Y | Y | Y | Y | - | Y | Y | Y | Y | Y | Y | Y | Y | Y | - | Y | - | - | - | Y | - | Y | Y | Y | - | Y | **21** | |
| 1. Presence of non-participants? | - | - | - | - | - | Y | Y | Y | - | - | - | - | - | - | Y | - | - | - | - | - | - | - | Y | - | Y | - | - | - | **6** | |
| 1. Description of sample given? | Y | Y | Y | Y | Y | Y | Y | - | Y | Y | Y | Y | - | Y | Y | Y | Y | Y | - | - | - | Y | Y | Y | Y | Y | Y | Y | **23** | |
| ***Data collection*** | | | | | | | | | | | | | | | | | | | | | | | | | | | | | | |
| 17. a) Interview guide provided | - | - | Y | - | - | Y | - | Y | - | - | - | Y | - | - | Y | - | Y | Y | - | Y | - | Y | Y | Y | - | Y | Y | - | **13** | |
| 1. b) Interview guide pilot tested? | Y | Y | - | - | Y | Y | - | - | - | - | - | - | - | - | Y | - | - | - | Y | Y | - | - | - | - | - | - | - | - | **7** | |
| 1. Reporting whether repeat interviews done? | - | - | - | - | - | Y | - | Y | Y | Y | Y | - | - | - | Y | Y | - | - | - | - | - | Y | Y | Y | Y | - | Y | - | **12** | |
| 1. Audio or visual recording | Y | Y | Y | Y | Y | Y | Y | Y | Y | - | Y | Y | Y | - | Y | Y | Y | Y | Y | Y | Y | Y | Y | Y | Y | Y | - | Y | **25** | |
| 1. Field notes kept? | Y | Y | Y | - | Y | Y | - | - | Y | Y | - | Y | Y | - | Y | Y | Y | - | Y | Y | Y | - | - | - | Y | - | Y | - | **17** | |
| 1. Duration of interviews or focus groups reported? | Y | Y | - | Y | Y | Y | Y | - | Y | - | Y | Y | - | - | Y | Y | Y | Y | Y | - | - | Y | Y | Y | Y | Y | - | - | **19** | |
| 1. Data saturation discussed? | Y | - | - | - | - | Y | - | - | Y | - | Y | Y | - | - | - | - | Y | Y | - | - | - | - | - | - | - | - | - | - | **7** | |
| 1. Transcripts returned to participants? | - | Y | - | - | Y | - | - | Y | - | - | - | - | - | - | - | - | - | - | Y | - | - | Y | Y | Y | - | - | - | - | **7** | |
| **Domain 3: analysis and findings *- Data analysis*** | | | | | | | | | | | | | | | | | | | | | | | | | | | | | | |
| 1. Number of data coders reported | Y | - | Y | - | - | - | Y | Y | - | Y | - | Y | Y | - | - | Y | Y | Y | - | Y | - | - | - | - | Y | Y | - | - | **13** | |
| 1. Description of the coding tree provided? | - | - | - | - | - | Y | - | Y | - | - | - | Y | Y | - | - | - | - | Y | - | Y | Y | - | - | - | - | - | - | - | **7** | |
| 1. Derivation of themes reported – in advance or from the data? | Y | Y | Y | Y | Y | - | Y | Y | Y | - | Y | Y | Y | - | Y | Y | Y | Y | Y | Y | Y | Y | Y | - | Y | - | Y | Y | **23** | |
| 1. Use (or not) of software management of data reported | Y | - | - | - | - | - | - | - | - | Y | - | - | - | - | Y | - | - | - | - | - | - | - | Y | - | - | - | - | Y | **5** | |
| 1. Participant checking of findings? | - | Y | - | - | Y | Y | - | Y | Y | - | - | - | - | - | - | - | - | - | - | - | - | Y | Y | Y | Y | - | - | Y | **10** | |
| ***Reporting*** | | | | | | | | | | | | | | | | | | | | | | | | | | | | | | |
| 1. Quotations presented and identified | Y | Y | Y | Y | - | Y | Y | Y | Y | - | Y | Y | Y | Y | Y | Y | Y | Y | Y | Y | Y | Y | Y | Y | Y | Y | Y | Y | **26** | |
| 1. Data and findings consistent | Y | Y | Y | Y | Y | Y | Y | Y | Y | Y | Y | Y | Y | Y | Y | Y | Y | Y | Y | Y | - | Y | Y | Y | Y | Y | Y | Y | **27** | |
| 1. Clarity of major themes | Y | Y | Y | Y | Y | Y | Y | Y | Y | Y | Y | Y | Y | Y | Y | Y | Y | Y | Y | Y | Y | Y | Y | Y | Y | Y | Y | Y | **28** | |
| 1. Clarity of minor themes – diverse cases or discussion of minor themes presented? | - | Y | Y | - | Y | - | Y | Y | Y | Y | Y | - | Y | Y | Y | Y | Y | Y | Y | Y | Y | Y | Y | Y | Y | Y | Y | Y | **24** | |
