# Supplementary material for: Complementary medicine products used in pregnancy and lactation and an examination of the information sources accessed pertaining to maternal health literacy: a systematic review of qualitative studies
Source: BMC Complement Altern Med. 2018 Jul 31;18:229. doi: 10.1186/s12906-018-2283-9 (PMC6069845; doi:10.1186/s12906-018-2283-9)
Supplement: Supplementary file 3 — Information sources accessed by women using CMPs in pregnancy and lactation by country groupings. (DOCX 105 kb) [file 12906_2018_2283_MOESM3_ESM.docx]

# Additional file 3: Information sources accessed by women using CMPs in pregnancy and lactation by country groupings

#### Figure A1: Information sources accessed by women using CMPs in pregnancy and lactation by country groupings.

*LIC = low income economy; LMIC = lower middle income economy; UPIC = upper middle income economy; HIC = high income economy according to The World Bank Classifications (2017), based on 2015 gross national income per capita*.

## Table A1: Information sources accessed by women by country groupings

| **Information source** | **Number of papers** | **Countries with reference** |
| --- | --- | --- |
| Shared cultural knowledge / traditions | 14 papers | - Tanzania (LIC) (Young & Ali, 2005) - Uganda (LIC) (Rutakumwa & Krogman, 2007; Waiswa et al., 2008) - Ghana (LMIC) (Aborigo et al., 2012) - Indonesia (LMIC) (Damanik, 2009; Wulandari & Whelan, 2011) - Morocco (LMIC) (Obermeyer, 2000) - Nigeria (LMIC) (Ejidokun, 2000) - Swaziland (LMIC) (Thwala et al., 2011) - Chinese women in China (UPIC), Taiwan (HIC) and USA (HIC) (Callister et al., 2011) - Thailand (UPIC) (Liamputtong et al., 2005; Elter et al., 2015) - Punjabi women in Canada (HIC) (Grewal et al., 2008) - Japanese women living in the USA (HIC) (Yeo et al., 2000) - Hmong women in Australia (HIC) but from a LMIC (Rice, 2000) |
| Women Elders - Mothers, mothers in law and grandmothers, other experienced female family members | 12 papers | - Tanzania (LIC) (Juntunen et al., 2000) - Uganda (LIC) (Rutakumwa & Krogman, 2007) - Ghana (LMIC) (Aborigo et al., 2012) - Indonesia (LMIC) (Damanik, 2009; Wulandari & Whelan, 2011) - Swaziland (LMIC) (Thwala et al., 2011) - South Africa (UPIC) (Mogawane, Mothiba, & Malema, 2015; Ngomane & Mulaudzi, 2012) - Thailand (UPIC) (Elter et al., 2016; Rice, 2000) - Chinese women in China (UPIC), Taiwan (HIC) and USA (HIC) (Callister et al., 2011) - Punjabi women in Canada (HIC) but from a LMIC (Grewal et al., 2008) |
| Traditional Birth Attendants or Traditional Midwives | 11 papers | - Tanzania (LIC) (Juntunen et al., 2000; Young & Ali, 2005) - Uganda (LIC) (Rutakumwa & Krogman, 2007; Waiswa et al., 2008) - Ghana (LMIC) (Aborigo et al., 2012; Dako-Gyeke, Aikins, Aryeetey, McCough, & Adongo, 2013) - Morocco (LMIC) (Obermeyer, 2000) - Nigeria (LMIC) (Okafor et al., 2014) - Lao People's Democratic Republic (LMIC) (Lamxay et al., 2011) - South Africa (UPIC) (Mogawane et al., 2015; Ngomane & Mulaudzi, 2012) |
| Biomedical health care practitioners (nurses, midwives, doctors) | 9 papers | - Tanzania (LIC) - Zanzibar (Young & Ali, 2005) - Ghana (LMIC) (Wilkinson & Callister, 2010) - Nigeria (LMIC) (Ejidokun, 2000) - Australia (HIC) (Sim et al., 2014) - Canada (HIC) (Westfall, 2003a, 2003b) - Japanese women living in the USA (HIC) (Yeo et al., 2000) - United Kingdom (HIC) (Holst et al., 2009; Warriner et al., 2014) |
| Family and friends in general (outside mothers in law and elders) | 9 papers | - Indonesia (LMIC) (Wulandari & Whelan, 2011) - Lao People's Democratic Republic (LMIC) (Lamxay et al., 2011) - South Africa (UPIC) (Ngomane & Mulaudzi, 2012) - Australia (HIC) (Sim et al., 2014) - Canada (HIC) (Westfall, 2003a, 2003b) - Japanese women living in the USA (HIC) (Yeo et al., 2000) - United Kingdom (HIC) (Holst et al., 2009; Warriner et al., 2014) |
| Traditional (non-Western) herbalists or healers | 8 papers | - Ghana (LMIC) (Aborigo et al., 2012; Dako-Gyeke et al., 2013; Wilkinson & Callister, 2010) - Morocco (LMIC) (Obermeyer, 2000) - Swaziland (LMIC) (Thwala et al., 2011) - Tanzania (LIC) (Juntunen et al., 2000) - South Africa (UPIC) (Mogawane et al., 2015) - Hmong women in Australia (HIC) but from a LMIC (Rice, 2000) |
| Western CAM HCPs (Western herbalists, homoeopaths or naturopathic practitioners) | 5 papers | - Australia (HIC) (Sim et al., 2014) - Canada (HIC) (Westfall, 2003a, 2003b) - United Kingdom (HIC) (Holst et al., 2009; Warriner et al., 2014) |
| Internet | 3 papers | - Australia (HIC) (Sim et al. 2014) - Canada (HIC) (Westfall, 2003b) - United Kingdom (HIC) (Holst et al., 2009) |
| Husbands | 4 papers | - Indonesia (LMIC) (Damanik, 2009; Wulandari & Whelan, 2011) - Punjabi women in Canada (HIC) (Grewal et al., 2008) - Japanese women in the USA (HIC) (Yeo et al., 2000) |
| Medicine women, magical healers or shamans | 2 papers | - Thailand (UPIC) (Liamputtong et al., 2005) - Hmong women in Australia (HIC) but from a LMIC (Rice, 2000) |
| Spiritualists or religious leaders | 2 papers | - Ghana (LMIC) (Dako-Gyeke et al., 2013) - South Africa (UPIC) (Mogawane et al., 2015) |
| Own knowledge or intuition | 1 paper | - Canada (HIC) (Westfall, 2003b) |
| Lactation consultants | 1 paper | - Australia (HIC) (Sim et al., 2014) |
| Herbal shops | 1 paper | - Canada (HIC) (Westfall, 2003b) |
| Books written by midwives, herbalists, childbirth educators | 1 paper | - Canada (HIC) (Westfall, 2003b) |
| Male heads of households | 1 paper | - Ghana (LMIC) (Aborigo et al., 2012) |
