# Supplementary material for: Complementary medicine products used in pregnancy and lactation and an examination of the information sources accessed pertaining to maternal health literacy: a systematic review of qualitative studies
Source: BMC Complement Altern Med. 2018 Jul 31;18:229. doi: 10.1186/s12906-018-2283-9 (PMC6069845; doi:10.1186/s12906-018-2283-9)
Supplement: Supplementary file 4 — Full thematic analysis - perceived benefits of CMP use in different stages of the childbearing continuum. (DOCX 54 kb) [file 12906_2018_2283_MOESM4_ESM.docx]

# Additional file 4: Full thematic analysis - perceived benefits of CMP use in different stages of the childbearing continuum

| **Perceived benefit** | **Type of CMP as identified by the authors** | **Country and reference** | **Selected quotes**  *(in italics – participant direct quotes;* in Roman (non-italicised) - text quotes (the papers did not always include quotes) |
| --- | --- | --- | --- |
| 1. **Pregnancy** | | | |
| ***Women’s use of CMPs in pregnancy for the benefit of the pregnancy – perceived physical benefits*** | | | |
| Prevention of vaginal bleeding and miscarriage in early pregnancy | Herbal medicines (ingested) | - Tanzania (Juntunen et al., 2000) - South African (Ngomane & Mulaudzi, 2012) - Ghana (Dako-Gyeke et al. 2013) | *“At the initial stages of my pregnancy I was bleeding and I came to the hospital for drugs but it was persistent. So I went for herbal medicine and it helped me” (Focus group participant, ANC client, Madina)”* (Dako-Gyeke et al. 2013, p211)  *“A herbal medicine, Mpundulo, was given by a midwife for me to drink to prevent abortion.”* (Ngomane & Mulaudzi, 2012, p35.) |
|  | Herbal medicines (ingested and external applications) | - South African (Ngomane & Mulaudzi, 2012) | *“After I was sure that I was pregnant, a grass thread (Ritlangi) was made to prevent abortion. …The same grass is boiled [and the mixture drunk] to ensure a safe pregnancy.”* (Ngomane & Mulaudzi, 2012, p35.) |
| Protect against vaginal leaking and bleeding in both early and late pregnancy | Herbal medicines (ingested) | - Tanzania (Juntunen et al., 2000) | “ ‘Taking local herbs’ is important in the different phases of pregnancy. Expecting mothers usually go to the healer to get local herbs because in early pregnancy the main concern is to prevent the vaginal bleeding heralding miscarriage ... [late in their pregnancies] they start taking local herbs to protect against vaginal leaking or bleeding” (Juntunen et al., 2000, p179). |
| Ensure a safe pregnancy | Herbal medicines (ingested) | - South African (Ngomane & Mulaudzi, 2012) | *“I have been advised to drink boiled herbs (Mbita) for the preservation and protection of my unborn baby, so that I may have a safe pregnancy and labour.”* (Ngomane & Mulaudzi, 2012, p34)  *“Ritlangi [a type of runner grass] was cooked and I had to drink the water from the concoction. …The boiled grass is tied around the waist to strengthen my pregnancy.”* (Ngomane & Mulaudzi, 2012, p33) |
| ***Women’s use of CMPs in pregnancy for the benefit of the baby – perceived physical benefits*** | | | |
| Promotion of the developing baby’s physical health - assist the baby’s intrauterine growth and support their well-being, health and vitality | Herbal medicine (ingested) | - Swaziland (Thwala et al., 2011) - Ghana (Dako-Gyeke et al., 2013) - South Africa (Ngomane & Mulaudzi, 2012) - Bali (Wulandari & Whelan, 2011) - Thailand (Liamputtong et al. 2005) | *“Siwasho also helps the unborn baby in the womb to grow well.”* (Thwala et al., 2011, p95)  *“I think both [iron pills and herbal medicine] are important, aren’t they? I take the herbals regularly and I feel that my baby is healthy that was also what I did in my first pregnancy. I regularly took the herbals and nothing’s wrong with my baby. In fact, he was very vigorous. (Woman 6)”* (Wulandari & Whelan, 2011, p868-9)  *“It (herbal medicine) makes the baby strong and healthy and you don’t experience pain as compared to the hospital drugs” (Focus group participant, TBA Client, Kwabenya)* (Dako-Gyeke et al., 2013, p211)  “The pregnancy needs to be strengthened with herbs to prevent malformation of the fetus and a miscarriage” (Ngomane & Mulaudzi, 2012, p33)  “Consumption of traditional herbal medicine was also mentioned as a way of preparing for an easy birth. The traditional herbal medicine was referred to as *ya tom*. A woman must consume *ya tom* three times per day for three consecutive days. Women can purchase dried herbal medicine and boil it until it reduces to small cup quantity and drink it as tea. This is believed to make the baby strong, hence facilitating an easy birth.” (Liamputtong et al. 2005, p146) |
|  | Prenatal vitamins, folic acid, iron supplements | - Ghana [37] | “Most women took advantage of health care services and recommendations:  *‘When I was pregnant I visited the hospital regularly; I made sure that I ate good, nutritious food; and also I took all the drugs [vitamins] that were given to me at the clinic.’ ”* (Wilkinson & Callister, 2010, p211) |
|  | Iron tablets | - Indonesia (Bali) (Wulandari & Whelan, 2011) | *“Sometimes when he [husband] ask me to take the iron tablets and explain that it’s for the sake of the baby’s health, that’s when I thought that I have to take the tablets. (Woman 8)”* (Wulandari & Whelan, 2011, p870) |
| Monitor the baby’s health and growth | Herbal medicines (external applications) | - South Africa (Ngomane & Mulaudzi 2012) | *“This grass tied around my abdomen will help me to observe if the pregnancy is growing. …The grass thread around my waist will become tighter and tighter and this shows that the fetus is well and growing”* (Ngomane & Mulaudzi 2012, p33 & 35) |
| No perceived benefit for the use of CMPs in pregnancy – taking vitamins was incompatible with Japanese cultural beliefs around taking medications in pregnancy | Prenatal vitamins | - Japanese women living in the USA (Yeo et al. 2000) | *“I have been eating Japanese food in the United States just like I did in Japan when I had my first child. I never took a vitamin with my first child . . . and it did not have any bad effects on my child . . . then American doctors told me that it’s better to take vitamins . . . I don’t mind taking it, but I don’t know why I need to take it, as nothing bad happened with my first child in Japan.”* (Yeo et al., 2000, p194) |
| ***Women’s use of CMPs in pregnancy for the benefit of the mother – perceived physical benefits*** | | | |
| Prevention or treatment of common illnesses associated with pregnancy like thrush and urinary tract infections | Herbal medicines (ingested) | - Swaziland (Thwala et al., 2011) - Nigeria (Okafor et al. 2014) - Canada (Westfall 2003a) | *“The importance of tiwasho [medicinal potion of religious origin] is to assist you as a pregnant woman in any health problem that may arise during the pregnancy”* (Thwala et al., 2011, p95 & 101)  “The participants identified ‘*aseje’*, (a special concoction, mainly herbs) as one of the attractions of seeking care from TBAs. It is believed that the ‘*aseje’* prevents development of any complications during pregnancy and labour and keeps pregnant women healthy” (Okafor et al. 2014, p46)  “[T]onics - which have no equivalent in biomedicine - were used by 23 women (85%) as a preventative health measure” (Westfall 2003a, p 29).  From Westfall’s (2003a) Table 7 (p28) the following herbs were used by participants to treat or prevent thrush and urinary tract infections:   - Coconut oil Cocos nucifera L. [Arecaceae] - supplies caprylic acid, suppresses yeast growth - Cranberry fruit *Vaccinium macrocarpon* Ait.[Ericaceae] - prevents bacterial adhesion to bladder wall |
| Prevention or treatment of non-pregnancy related illnesses | Herbal medicines (ingested) | - Canada (Westfall 2003a) - Uganda (Waiswa et al. 2008) | From Westfall’s (2003a) Table 7 (p28) the following herbs were used by participants to treat or prevent common illnesses:   - Echinacea *Echinacea* *spp*. - Immune stimulant - Fenugreek seed *Trigonella foenicum-graecum* L. [Fabaceae] – decongestant - Garlic clove *Allium sativum* L. [Liliaceae] - Anti-fungal agent   “Other cases where herbs are used [in pregnancy] included malaria and syphilis treatment” (Waiswa et al. 2008, p21) |
|  | Herbal medicines (external applications) | - South Africa (Ngomane & Mulaudzi, 2012) | *“The little knots tied along the ring of grass are a herb, Ndzenga [name of a tree] to help me not to be sick during pregnancy.”* (Ngomane & Mulaudzi, 2012, p35) |
| First line treatment of maternal danger signs in pregnancy | Herbal medicines (not detailed whether ingested or external applications) | - Uganda (Waiswa et al. 2008) | “Participants were able to identify some maternal danger signs including high fever (*omusudha*), severe pre-eclampsia (*amakiro*), hemorrhage (*okuva omusayi*), abdominal pain (*okulumwa munda*), and swelling of feet (*ebigere okuzimba*). … We found that in the community, danger signs are usually first treated with herbs, and they only seek medical care when the condition becomes worse.” (Waiswa et al. 2008, p21) |
| Protection against the development of pregnancy complications | Herbal medicines (not detailed whether ingested or external applications) | - Nigeria (Okafor et al. 2014) | “The participants identified ‘*aseje’*, (a special concoction, mainly herbs) as one of the attractions of seeking care from TBAs. It is believed that the ‘*aseje’* prevents development of any complications during pregnancy and labour and keeps pregnant women healthy. […]  *“Most women prefer TBA because they give them “aseje”* (Okafor et al. 2014, p46) |
| Safe support for mother’s own physical health | Herbal medicines (ingested) | - Indonesia (Bali) (Wulandari & Whelan, 2011) - Ghana (Dako-Gyeke et al. 2013) - Canada (Westfall 2003a) | *“Traditional tamarind and turmeric do no harm to our baby. In fact, it makes both of us healthier”… “My mother, my grandmother, my great grandmother had used this herbal to make us healthier. And it works, without any side-effects (Woman 7)”* (Wulandari & Whelan, 2011, p869)  *It (herbal medicine) gives strength, the hospital drug weakens me when I take them but the traditional medicines make me active (Focus group participant, TBA Client, Kwabenya)* (Dako-Gyeke et al. 2013, p211)  “Tonic herbs can be thought of as lying somewhere in between food and drugs; they are used therapeutically, to treat sub-clinical conditions or to prevent health degeneration. They are used to strengthen, nourish and support the body, to prevent rather than cure disease […] Tonic herbs were by far the most widely used herbal medicines (Table 7). Twenty-four of the 27 women used at least one of the nine tonic and nutritional herbs inventoried. The most popular herb was raspberry leaf *(Rubus idaeus)* - a uterine tonic - used by 22 women.” (Westfall 2003a, pp26-27). |
|  | Vitamins, minerals, homoeopathic remedies and herbal supplements (CAM) available over the counter | - UK (Warriner et al., 2014) | “Certainly for the women interviewed in this study, CAM provided a way of investing in their bodies, not just to prevent ill-health but as a way of fulfilling and optimising potential […] *‘I'd use complementary therapies before I resorted to drugs from the doctor, that's not to say I wouldn’t listen to the doctors if I was really sick. CAM are just more natural and most of the time you can keep yourself well by using natural things and eating well and generally looking after yourself’.(P8)”* (Warriner et al., 2014, p141). |
| Treatment of maternal anaemia; provision of extra nourishment | Herbal medicines (ingested); *Chomwe -* traditional iron supplements (ingested) | - Tanzania (Zanzibar) (Young & Ali, 2005) | *“*Another food-based treatment for anaemia is *uwatu* [*Trigonella Foenum-raecum* or fenugreek)]… Fenugreek is considered very nourishing for people in need of a dietary boost, such as the elderly or pregnant. Sometimes it is sprinkled directly into food, other times it is mixed with *samli ya ngombe* (ghee made of cow’s milk), garlic, milk, honey and several other untranslatable ingredients, such as *habasoda* and *zamda* to make *haluwat saumu*, a treatment for *baridi yabisi*, yet another type of anaemia.” (Young & Ali, 2005, pp53-54).  “Drinking the dark red water that results from boiling avocado leaves *(maji ya mapaya)* is believed to be another way of fortifying a person’s blood.” (Young & Ali, 2005, p54).  “*Chomwe* is a fragrant traditional medicine used to treat *safura*, one of the ‘anaemias’ in Pemba. It is made from powdered iron filings and *dawa za kikaango* ‘medicine of the clay pot’…” (Young & Ali, 2005, p54).  *“A pregnant woman should use a small teaspoonful of a mix of sanamaki (dried crumbled leaves with laxative properties), zamda (a minty, opaque, crystal-like substance that dissolves in water, obtained in Oman), turmeric, and chomwe once a day.”* (Young & Ali, 2005, p54) |
| Safe form of treatment for nausea and vomiting of pregnancy | Herbal medicines | - Canada (Westfall 2003a; Westfall 2004) | “Herbal remedies used to treat pregnancy-induced nausea and vomiting included ginger (*Zingiber officinale* Roscoe [Zingiberaceae]; used by six women), peppermint (*Mentha piperita* L. [Lamiaceae]; used by six women), and Cannabis (*Cannabis sativa* L. Cannabaceae]; used by one woman) […] Of the six women who used ginger, all considered it to be only moderately effective, if at all. […] The six women who used peppermint all found it to be somewhat helpful in soothing nausea. In every case, the herb was prepared as a tea, sometimes in combination with other herbs such as raspberry leaf (n=3). As noted above, three of the six women who used peppermint were also using ginger. Peppermint reportedly had a calmative effect on the stomach, in addition to reducing nausea, and its smell was not off-putting to any of the women. […] Unlike ginger and peppermint, Cannabis was not ingested; rather, the woman kept a small supply of the dried herb and a little pipe to smoke it in” (Westfall 2004, p32)  “Sixteen of the women (59%) expressed caution when asked how they felt about using herbal medicines while pregnant. All 27 women made it clear that they paid close attention to what they exposed themselves to while pregnant, for fear of harming the baby. … No women expressed concern over whether herbal medicines–as a general category–were safe to use in pregnancy; their concerns were around specific herbs, or around using any sort of medication, natural or synthetic. All the women felt that the herbs they used themselves were safe” (Westfall 2003a, p29) |
| Treatment of abdominal pain in pregnancy | Herbal medicines (ingested / external applications not detailed) | - Uganda (Rutakumwa & Krogman, 2007) | *“During pregnancy I used to have pain in my abdomen. The medication I was given at the clinic did not help and the problem persisted. I had to resort to traditional medicine, which helped.”* (Rutakumwa & Krogman, 2007, p117) |
| ***Women’s use of CMPs in pregnancy for the labour and birthing processes – perceived benefits*** | | | |
| Prevention of vaginal tearing during birth and reducing risk of caesarean section | Herbal medicines (external applications) | - Uganda (Rutakumwa & Krogman, 2007) | “A typical example is what is locally known as *amalagala*, a product of crushed sweet-potato leaves mixed with water. This mixture is administered to pregnant women, who bathe in it or sit on it to lessen the risk of requiring a Caesarean section or of vaginal tearing during delivery. The women did not discuss trial and error for this concoction but unanimously reported confidence in its efficacy” (Rutakumwa & Krogman, 2007, p117) |
| Prevention of foetal distress | Herbal medicines (ingested) | - South Africa (Ngomane & Mulaudzi, 2012) | *“In preparation for labour, Mbheswana [name of an herb] will be boiled to enhance labour and prevent fetal distress.”* (Ngomane & Mulaudzi, 2012, p33) |
| Use of herbal tonics to tone the uterus and strengthen it in preparation for labour | Herbal medicines (ingested) | - Canada (Westfall 2003a) | “In the contemporary Western approach to herbal medicine, tonic herbs are usually thought of in terms of their specific biological or nutritional function. For instance, a uterine tonic is a (sic) herb that strengthens uterine muscle by stimulating mild muscular contractions. Raspberry leaf is an example of a uterine tonic. […]The most popular herb was raspberry leaf (*Rubus idaeus*) - a uterine tonic - used by 22/27 women.” (Westfall 2003a, p26) |
| Prepare for an easy birth | Herbal medicines (ingested) | - Thailand (Liamputtong et al. 2005) - Lao People's Democratic Republic (Lamxay et al., 2011) - South Africa (Mogawane et al., 2015) | *“I ate a lot of pak plang during pregnancy. Pak plang is a soft vegetable and we make into hot soup, a Northern style hot soupy We eat all the leaves and the water because it is slippery and this makes the baby slip out easily.”* (Liamputtong et al. 2005, p146)  “Consumption of traditional herbal medicine was also mentioned as a way of preparing for an easy birth. The traditional herbal medicine was referred to as *ya tom*. A woman must consume *ya tom* three times per day for three consecutive days. Women can purchase dried herbal medicine and boil it until it reduces to small cup quantity and drink it as tea. This is believed to make the baby strong, hence facilitating an easy birth.” (Liamputtong et al. 2005, p146)  “Use of medicinal plants, either in steam-baths or consumed in decoctions, is avoided during pregnancy. The only advice reported was dietary, and recommended daily consumption of banana plant pseudostems or young leaf-sheaths (*Musa acuminata Colla*), as it would guarantee an easy delivery” (Lamxay et al. 2011, p14)  “[P]articipants had knowledge related to important reasons for adhering to their traditional medicine in relation to their conditions.  A participant said: *‘… [S]o that you will not be delayed when you give birth to the baby. It also makes the process of giving birth less painful.’* (Mogawane et al., 2015, p 5 of 8) |
|  | Herbal medicines (external applications) | - Thailand (Liamputtong et al. 2005) - South Africa (Ngomane & Mulaudzi, 2012) and (Mogawane et al., 2015) | “Another common practice is to gather *pak plang* and *maiyarab* plant (another vine-liked green plant) and make them into a loop and then boiled with water. A pregnant woman then showers with this herbal water. When she is taking a shower, the loop will be put on her head. This is believed to facilitate an easy birth.” (Liamputtong et al. 2005, p146)  *“When it is time to give birth, I will untie the knots in the grass belt around my waist and scatter the leaves of herbs all over my yard so as to have an easy and quick delivery.”* (Ngomane & Mulaudzi, 2013, p35) |
| Enhance or induce labour | Herbal medicines (ingested) | - South Africa (Ngomane & Mulaudzi, 2012) - Punjabi women in Canada (Grewal et al., 2008) - Tanzania (Juntunen et al. 2000) - Canada (Westfall, 2003a healing) - Morocco (Obermeyer, 2000) | *“When the time comes I will drink Mbheswana to start fast labour and prevent fetal distress.”* (Ngomane & Mulaudzi, 2012, p35)  *“Xirheti or Xiveve [an indigenous oxytocin] is the name of the herb that is boiled to drink to accelerate labour.”* (Ngomane & Mulaudzi, 2012, p35)  “Later in the pregnancy, some women also received specific foods from relatives to encourage the onset of delivery. For example, one woman shared her experience of receiving soonf (fennel seeds) roasted in brown sugar from her mother-in-law, a food that is thought to *“assist in sharpening [inducing] the [labor] pains”* (Grewal et al., 2008, p292)  “Taking local herbs is common when the expected time of giving birth approaches […] Mothers expect the labor to be fast and easy. Many women still fear caesarean section because a Bena man is allowed, if he so chooses, to divorce a wife who is *“so weak that she cannot even push the baby out.”* Because of all these reasons, they turn to a *waganga*, an older woman who knows how to hasten the labor by using certain local herbs. A mother who refuses to take local oxytocins is often given them secretly in tea or porridge by her mother-in-law or mother” (Juntunen et al., 2000, p179)  “As a general rule, the women expressed a greater comfort level with using herbs than pharmaceutical drugs. This was apparent from their choice to use herbs first, and only to use pharmaceutical drugs after herbal treatment failed (which happened during pregnancy in two instances, and for labour induction in two cases).”  From Westfall’s (2003a) Table 7 (p28) the following herbs were used by participants to induce labour:   - Blue cohosh *Caulophyllum thalictroides* (L.) Michx. [Berberidaceae] - Uterine stimulant - Castor bean oil *Ricinus communis* L. [Euphorbiaceae] - Uterine stimulant - Evening primrose oil Oenothera biennis L. [Onagraceae] - Cervical ripening agent   “When labor begins, women fumigate their lower body with burning incense, and they drink an infusion of milk and habb arrshad (peppergrass seeds) that is believed to heat them inside” (Obermeyer, 2000, p185) |
| Relieve labour pains | Herbal medicines (ingested) | - South Africa (Ngomane & Mulaudzi, 2012) - Ghana (Dako-Gyeke et al., 2013) | *“The traditional birth attendant gave me Xirhakarhani [a traditional analgesic] to alleviate excessive labour pains.”* (Ngomane & Mulaudzi, 2012, p35)  *“Roots of Xirhakarhani are boiled and the water is drunk to relieve excessive labour pains.” … ‘”Xirhomberhombe leaves are mixed with Dinda to accelerate labour and prevent severe pain.”* (Ngomane & Mulaudzi, 2012, p35)  *“It (herbal medicine) makes the baby strong and healthy and you don’t experience pain as compared to the hospital drugs” (Focus group participant, TBA Client, Kwabenya)* (Dako-Gyeke et al., 2013, p211) |
|  | Herbal medicines (ingested and external applications), vitamin pills | - Morocco (Obermeyer, 2000) | “Often in a home birth, the woman in labor may lie in bed instead of squatting on the floor as is the traditional practice, and some traditional birth attendants give vitamin pills or injections or administer pain relievers in the form of suppositories in addition to using herbal remedies” (Obermeyer, 2000, p190). |
| Induce expulsion of retained placenta | Herbal medicines (ingested) | - South Africa [76] - Hmong women in Australia (Rice 2000) | *“If the placenta is retained, Dinda is boiled and drunk to induce contractions”* (Ngomane & Mulaudzi, 2012, p35)  “White pepper contains `hot' property, so it helps to induce childbirth blood and left-over placenta in the uterus and makes the womb clean. This also helps in the relief of afterbirth pain:  *“The first meal is made with an egg and pepper, then after that you eat hot rice and [chicken] meat. . . We must do that so that it will stop the stomach pain. The truth is that the pepper is the most important. The pepper will help to wash your body so that it won't give you afterbirth pain.”* (Rice 2000, p25) |
|  | Herbal medicines (external applications) | - South Africa (Ngomane & Mulaudzi, 2012) | *If placenta birth is delayed, Rihlanga [two slender river canes] are used to pull it out manually”* (Ngomane & Mulaudzi, 2012, p35) |
| Relieve afterbirth pains | Herbal medicines (ingested) | - Hmong women in Australia (Rice 2000) | “White pepper contains `hot' property, so it helps to induce childbirth blood and left-over placenta in the uterus and makes the womb clean. This also helps in the relief of afterbirth pain:  *“The first meal is made with an egg and pepper, then after that you eat hot rice and [chicken] meat. . . We must do that so that it will stop the stomach pain. The truth is that the pepper is the most important. The pepper will help to wash your body so that it won't give you afterbirth pain.”* (Rice, 2000, p25)  “It is believed that afterbirth pains occur because the body is not clean from the childbirth blood and remaining placenta in the body: *“If it is because of her body, because it is not clean then we give her herbs [to eat] to wash her body. . . You must use herbs to wash it out.”* (Rice, 2000, p29) |
| ***Women’s use of CMPs in pregnancy to protect against spiritual threats to themselves and their unborn babies – perceived benefits*** | | | |
| Protect the baby from spiritual threats that could cause physical harm including death of the foetus or preterm labour | Herbal medicines (ingested) | - South Africa (Ngomane & Mulaudzi, 2012) and (Mogawane et al., 2015) - Swaziland (Thwala et al., 2011) | “All the women in this study stated that both the mother and baby might fall ill because of *kuhabula*. To prevent illness therefore, the women expressed belief in the power of traditional doctors and medicine, or divine prayer if the women or family was religious” *. . . [traditional medicines are taken] to make sure that the baby is protected on all fronts; protected from kuhabula [acquisition of illnesses from bad spirits in the environment] through the use of traditional medicine”* (Thwala et al., 2011, p95)  *“Some people practice the Kugata ritual [see external applications below] and drink herbal medicines called lipulede to protect the baby. I do not. I use the tindayela [a religious potion made from tea] which protect the baby from all these things.” ”* (Thwala et al., 2011, pp95-06)  “…pregnancy needed to be preserved physically and spiritually with herbs. It is believed that having contact with other women at the clinic may subject women to evil spirits who could harm the fetus. The pregnancy needs to be strengthened with herbs to prevent malformation of the fetus and a miscarriage, which could be inflicted by jealous people.” (Ngomane & Mulaudzi, 2012, p33)  *‘…[O]nce you become aware that you are pregnant, they will prepare for you for some solemn obligatory prescription (ditaelo) like Joko tea which is very weak so as to protect you from evil spirits and witchcraft.’* Furthermore, a participant indicated: *‘Yes, that you will always live with it and drink it to protect one to have abortion because of people who might be jealous.’* (Mogawane et al., 2015, p 3 of 8). |
|  | Herbal medicines (external applications) | - Swaziland (Thwala et al., 2011) - Tanzania (Zanzibar) (Young & Ali, 2005) | “*When pregnant, I drink traditional herbal medicines called timbita. I also do the kugata ritual [traditional medicinal ritual which involves making cuts on the skin and applying some traditional medicine onto the bleeding wound] for lipulede…” (Thwala et al., 2011, p96 & 101)*  “If protection fails, and one becomes ‘infected’ with spirits, the spirits that were causing anaemia could be treated with *kuchanjwa*. *Kuchanjwa* is a treatment performed by an *mganga* (traditional healer) whereby several small incisions are made on the forehead, near the armpits, the knees and the ankles. A paste of chicken blood, honey and secret roots is rubbed into the wounds. This process is purported to change the smell of the person’s blood [to protect against or expel blood-sucking spirits]” (Young & Ali, 2005, p55) |

| 1. ***Women’s use of CMPs during breastfeeding*** | | | |
| --- | --- | --- | --- |
| ***Women’s use of CMPs for the benefit of the breastfeeding process – physical benefits*** | | | |
| Increased breastmilk production – perceived and diagnosed milk insufficiency | Herbal medicines and vitamin tablets (ingested) | - Ghana (Aborigo et al. 2012) - Canada (Westfall 2003b) - Australia (Sim et al., 2014) | “Some mothers are given *‘local herbal concoctions to drink and some to massage the breast’* (IDI, Mother of newborn, supervised delivery) to stimulate the production of breast milk.” (Aborigo et al. 2012, p76)  *“I think it’s [fenugreek] worth trying. And as for me, I certainly find that useful and reassuring that I have found something effective to increase my milk supply. As a new mum, you just never know, you never know what is coming, what problems you will encounter and I certainly did not anticipate that milk supply will be an issue. I have always thought that breastfeeding is easy and will come naturally because everyone else does it, and I wasn’t told about it being an issue”. (BW 12).* (Sim et al., 2014, p216)  “Four of the participants used fenugreek seed as a galactagogue, and in each case, it was prepared as a tea … By and large, they considered it to be effective, though they were often unsure of how to measure its efficacy. As one woman said: *“I think [it’s working]*. *For a while there, I thought I wasn’t producing as much milk, but it could have just been my imagination.”* (Westfall, 2003b, p24) |
|  | Herbal medicines (external application) | - Ghana (Aborigo et al. 2012) - Canada (Westfall 2003b) | “Other methods of stimulating the production of breast milk include … massaging the breast with shea-butter” (Aborigo et al. 2012, p76)  “In this study, four women used fennel seed or oil, and all considered it to be an effective galactagogue … one decided to place the oil on her wrist instead, hoping to benefit from its odour.” (Westfall 2003b, p24). |
| Use of galactagogues ‘just in case’ breastmilk supply needs support | Herbal medicines (ingested) | - Canada (Westfall 2003b) - Australia (Sim et al., 2014) | “In this study, four women used fennel seed or oil, and all considered it to be an effective galactagogue. … Only two of these women had experienced milk supply problems; the other two were using the herb prophylactically.” (Westfall, 2003b, p24)  “Participants reported four main reasons for the use of herbal galactagogues, namely perceived insufficient milk supply, diagnosed insufficient milk supply, as a supplement and as part of the tradition. Besides those who had been diagnosed with insufficient milk supply by health professionals, all other participants embraced the “just-in-case” approach to use herbal galactagogues prophylactically in order to avoid breast milk supply issues” (Sim et al., 2014, p216) |
|  | Vitamin tablets (ingested) | - Japanese women living in the USA (Yeo et al., 2000) | “They [mothers] were willing to take vitamins postnatally, but only because their Japanese friends, rather than a physician or nurse, said that this supplementation helped increase the volume of breastmilk.” (Yeo et al., 2000, p194-5) |
| Use of galactagogues to build supply as part of a cultural tradition (note, no mention of perceived insufficiency) | Herbal medicines (ingested) | - Indonesia (Damanik, 2009) - Thailand (Elter et al. 2016) | *“For Bataknese women who have just given birth, particularly in the first month, they must consume torbangun soup so that they will produce a lot of breast milk. Our ancestors have handed down this tradition for more than hundred years.”* (Damanik, 2009, p67)  *“My breast milk production was abundant after consuming torbangun. My breast felt full and there were drops of breast milk around my nipples”* (Damanik, 2009, p70)  “The women mentioned a few herbs that made the drink smell good and sooth her emotion and spirituality. Hansa (HS) had fragrant herbs that promoted breast milk production. She said, *‘Mak khan hai (a name of a local plant) or mak tong lang (a name of a local plant) made it (hot water) smell good.’* (Elter et al., 2016, p254) |
| Use of galactagogues supports post-birth recovery and builds breastmilk supply | Herbal medicines (ingested) | - Indonesia (Damanik, 2009) - Punjabi women in Canada (Grewal et al., 2009) - Hmong women in Australia (Rice, 2000) - Kry people of Lao People's Democratic Republic (Lamxay, et al., 2011) | *“Torbangun portrays that a woman, who has just given birth loses a lot of energy, hence feels worn out. However, she must breastfeed the baby, therefore needs a lot of extra energy. She doesn’t only need the energy during the day when she is awake but also during the night when she has to breastfeed. This will go on for months during the breastfeeding period. And for the mother to be able to have the extra energy, she must consume torbangun soup. By consuming the soup she will be able to get up and stay awake at night and breastfeed the baby”* (Damanik, 2009, p68)  “During the early postpartum period as women recovered, family members again provided certain foods that were believed to have ‘hot effects’ and bring the body into balance. These types of food are seen as essential for healing and recovery from the birthing process (arising from Ayurveda traditions), including relieving back pain, promoting menstrual flow to cleanse the body, building the mother’s milk supply, and preventing weakness and illness in later life. ‘Hot foods’ included … chai (fennel seed tea with ginger) … and other special foods … made from ‘heat-producing’ ingredients such as ginger powder, fennel seeds … and special herbs.” (Grewal, 2008, p294)  “The Hmong rely heavily on their green herbs during confinement. Herbs play an important role in maintaining the health of a new mother. There are several common green herbs known as *tshuaj quib* (chicken herbal medicine) used in confinement. These herbs serve several purposes. These include relieving aches and pain after birth, producing extra blood after birth, washing out the childbirth blood and remaining placenta from the uterus, replacing energy and strength lost during birth, promoting a good appetite, improving weight lost during birth and enhancing breast milk” (Rice, 2000, p29)  “Starting the day following parturition the mother eats a special diet … complemented with cooked banana inflorescences *(Musa acuminata Colla)* and rattan shoots *(Calamus rudentum Lour.)* as lactagogue. In addition the mother will drink a variety of hot decoctions to aid in postpartum recovery [including] *Psychotria sarmentosa* Blume to aid healing of the perineum, retraction of the uterus, as a lactagogue, and for general postpartum recovery; a mixture of *Zingiber officinale* Roscoe and *Alpinia galanga* (L.) *Willd*. to protect and reduce postpartum fever or dizziness resulting from postpartum anaemia, and as a lactagogue; […] As during the first postpartum phase, a special diet is prescribed [in the second postpartum phase, complemented with cooked banana inflorescences (*Musa acuminata Colla*) and rattan shoots (*Calamus rudentum Lour*.), as a lactagogues […] [In the third postpartum phase] The same diet is eaten as during the second postpartum phase, with a continued focus on plant species that reportedly enhance breast-milk supply.” (Lamxay et al., 2011, p14) |
| ***Women’s use of CMPs in breastfeeding for the benefit of the breastfeeding baby – physical benefits*** | | | |
| Protection of the breastfeeding baby through mother’s use of CMPs | Herbal medicines (ingested) | - Tanzania (Juntunen et al., 2011) | “The ingestion of local herbs is used as a means of warding off any harmful effects to the baby that can be caused by either the father or the grandmother partaking in sexual relations. The Bena have a traditional belief that grandmothers are not expected to continue their sexual life. To protect the baby from health problems in such a case, all three parties - the newly delivered mother, her mother, and her mother-in-law - should take local drugs [herbal medicines] before the grandmother sees the baby for the first time” (Juntunen et al., 2011, p177) |
| Purification of mother’s breasts in preparation for breastfeeding and to ensure breastmilk is sweet | Herbal medicines (external applications) | - Ghana (Aborigo et al. 2012) | “ …first time mothers … are required to express their first milk into a container and put black ants in it to test for bitterness. If the ants succeed in crawling out, the milk is declared wholesome and the mother can go ahead and breastfeed. On the other hand, if the ants die, the breast milk is considered bitter, […] dirty and poisonous and can give the child diarrhea, which could lead to death. The mother must therefore go through a rite called *puure-nyoone* in Kasem and *wobi-biisa* in Nankani, to purify the milk before initiating breastfeeding. *Puure-nyoone* or *wobi-biisa* involves the use of herbs or shea-butter to rub or wash the breasts. …When a [first time mother] has gone through these rites, it is assumed that the breastmilk is no longer bitter and the mother can initiate breastfeeding” (Aborigo et al. 2012, p76) |
| Promotion of baby’s health through enabling mother to continue to breastfeed | Herbal medicines (ingested) | - Canada (Westfall, 2003b) - Australia (Sim et al,. 2014) | “All of the women who were interviewed held a philosophy of 'breast is best'; human milk was believed to be more wholesome than infant formula. Breast-feeding was considered to be […] beneficial for [the] baby: *““It's just amazing to see how much they thrive. Even while they're nursing, the expressions on his face, and the sounds he makes, you know, I can tell that it's really important for him to do that.”* (Westfall, 2003b, p24)  “All participants seemed to have adopted the ‘breast is best’ philosophy. These women acknowledged and appreciated the health, physical and psychological benefits of breastfeeding to both mothers and infants. […] Recognition of the importance and significance of breastfeeding was identified as the main facilitator to develop perseverance and a determined attitude to breastfeed:  *“I mean honestly, if drinking snake oil would make me have more breast milk I would have done it, anything that helps!”* (Sim et al., 2014, p216) |
| ***Women’s use of CMPs in breastfeeding for the benefit of the mother – physical benefits*** | | | |
| Expulsion of lochia through ‘uterine cleansing’ and control of postpartum bleeding | Herbal medicines (ingested) | - Indonesia (Damanik, 2009) - Hmong women in Australia (Rice 2000) - Punjabi women in Canada (Grewal et al., 2008) | *“I had my reproductive organs clean faster from the childbirth blood than my Javanese neighbour who didn’t consume torbangun soup. I told her to consume torbangun, but she didn’t want to.”* (Damanik, 2009, p70)  *“You eat them [chicken herbal medicine] so that your body will settle back to normal quicker and if you don't use them then it will take you a long time to get back to normal. The bleeding will go on for a long time and that will make you very thin. That is not good. . . If you bleed too long the body won't get back to normal again and this can make you pale and skinny. If you have the chicken herbs to eat then your blood will be good and you will feel strong quickly. . . You eat them to give you strength and also to wash out your blood quickly too”* (Rice, 2000, p29)  “It is believed that afterbirth pains occur because the body is not clean from the childbirth blood and remaining placenta in the body: *“If it is because of her body, because it is not clean then we give her herbs [to eat] to wash her body. . . You must use herbs to wash it out.”* (Rice, 2000, p29)  “During the early postpartum period as women recovered, family members again provided certain foods that were believed to have ‘hot effects’ and bring the body into balance. These types of food are seen as essential for healing and recovery from the birthing process (arising from Ayurveda traditions), including relieving back pain, promoting menstrual flow to cleanse the body, building the mother’s milk supply, and preventing weakness and illness in later life. ‘Hot foods’ included … chai (fennel seed tea with ginger) … and other special foods … made from ‘heat-producing’ ingredients such as ginger powder, fennel seeds … and special herbs.” (Grewal, 2008, p294)  “During the whole first day she will also drink a hot decoction of lightly roasted corn (*Zea mays* L.) to reduce abdominal pain and aid expulsion of lochia. Starting the day following parturition the mother […] will drink a variety of hot decoctions to aid in postpartum recovery (Table 1 & 2): *Tacca chantrieri* André to aid healing of the perineum, retraction of the uterus, expulsion of lochia, and reducing abdominal pain […] and a mixture of *Lagerstroemia calyculata* Kurz and *Choerospondias axillaris* (Roxb.) to aid healing of the perineum, retraction of the uterus, expulsion of lochia…” (Lamxay et al., 2011, p.14) |
| Assists in recovery after childbirth | Herbal medicines (ingested) | - Indonesia (Damanik, 2009) - Punjabi women in Canada (Grewal et al., 2008) - Kry people in Lao People's Democratic Republic (Lamxay et al., 2011) - Chinese women living in the People’s Republic of China, Taiwan and the USA (Callister et al., 2011) - Thailand (Elter et al., 2016) | *“Torbangun or tarbangun is Bataknese Simalungun language. The word ‘bangun’ means ‘wake up’. It means that the leaf has the benefit of lifting the spirit or giving more power/strength to the mother.”* (Damanik, 2009, p68)  *“After consuming torbangun soup, I felt fit and my tiredness wore off not too long after giving birth. The soup is good for recovery after giving birth.”* (Damanik, 2009, p70)  “During the early postpartum period as women recovered, family members again provided certain foods that were believed to have ‘hot effects’ and bring the body into balance. These types of food are seen as essential for healing and recovery from the birthing process [and] included chai (fennel seed tea with ginger), ginger powder, fennel seeds … and special herbs” (Grewal et al., 2008, p294)  “In addition the mother will drink a variety of hot decoctions to aid in postpartum recovery … [herbs] to aid healing of the perineum, retraction of the uterus, expulsion of lochia, and reducing abdominal pain, …[herbs] to protect and reduce postpartum fever or dizziness resulting from postpartum anaemia, and as a lactagogue; … and in case of postpartum secondary haemorrhage” (Lamxay et al., 2011)  “A woman living in the United States reported she used *yi mu cao* or Chinese motherwort for postpartum replenishment of her blood” (Callister et al., 2011, p392)  “Based on traditional medicine, rural postpartum Thai women believe that childbirth causes imbalance to their *self*, which is composed of body, mind–heart, and energy. Imbalance alters immunity; hence, the self is vulnerable to sickness. To heal mind–heart or spirit, with the help from her mother or mother-in-law, new mothers observe many postpartum practices [including hot herbal drinks and baths].” (Elter et al., 2016, p254) |
| Restoration of balance through heat | Herbal medicines (ingested) | - Chinese women living in the People’s Republic of China, Taiwan and the USA (Callister et al., 2011) | “ ’Doing the month’ includes activity restrictions, avoiding ‘wind chill’ … and dietary requirements such as eating chicken soup, pigs’ feet, and raw ginger soup with Chinese herbs to ‘rid the body of cold’ ” (Callister et al., 2011, p390)  “A woman living in the United States reported she used *yi mu cao* or Chinese motherwort for postpartum replenishment of her blood” (Callister et al., 2011, p392) |
|  | Herbal medicines (external applications) | - Hmong women in Australia (Rice, 2000) | “In the case of ill health arising from not keeping the body warm enough, the Hmong will resort to herbal medicines. The herbs are used in combination with hot rocks and this method is known as *tshuaj ntxhawb* - the steamed herbs method. Herbs are boiled and the rocks are heated up in a fire. Then the rocks are put into the hot herbs which creates hot steam. The mixture is placed near the body of the woman and a blanket is placed on her body. The heat is trapped inside the blanket and is absorbed by her body. This assists the woman to regain heat lost from her body and relieve her from any symptoms caused by coldness of the body” (Rice, 2000, p30). |
| Treatment of a prolapsed uterus | Herbal medicines (ingested and external applications) | - Hmong women in Australia (Rice, 2000) | *“[If] that [uterine prolapse] happens then there are herbal medicines that are crushed to put on that area. There are some herbal medicines to drink as well and that will move that thing back into the vagina”* (Rice, 2000, p26) |
| Protection of the mother’s future health | Herbal medicines (ingested) | - Punjabi women in Canada (Grewal et al., 2008) - Chinese women living in the People’s Republic of China, Taiwan and the USA (Callister et al., 2011) | “During the early postpartum period as women recovered, family members again provided certain foods that were believed to have ‘hot effects’ and bring the body into balance. These types of food are seen as essential for healing and recovery from the birthing process (arising from Ayurveda traditions), including relieving back pain, promoting menstrual flow to cleanse the body, building the mother’s milk supply, and preventing weakness and illness in later life. ‘Hot foods’ included … chai (fennel seed tea with ginger) … and other special foods … made from ‘heat-producing’ ingredients such as ginger powder, fennel seeds … and special herbs.” (Grewal et al., 2008, p294)  “Considered the most important Chinese cultural practice is ‘doing (or sitting) the month’ (*zuoyuezi*). … ‘Doing the month’ includes activity restrictions, avoiding ‘wind chill’ ... and eating raw ginger soup with Chinese herbs to ‘rid the body of cold’ … If such practices as described are not followed, the new mother is at risk for ‘the month disease,’ which is thought to have deleterious effects on their health for the rest of their lives (Callister et al., 2011, pp390-1) |
| ***Women’s use of CMPs during breastfeeding for the benefit of the mother – perceived mental-emotional benefits*** | | | |
| Increased self-confidence, self-empowerment and reassurance | Herbal medicines (ingested and external applications) | - Australia (Sim et al., 2014) - Canada (Westfall 2003b) | “Many participants also mentioned the feeling of reassurance through the use of herbal supplements during breastfeeding, which was especially important for first-time mothers. Hence, the use of herbal galactagogue was described as a method of reassurance in the context of their own perceptions. The positive emotional impact contributed to the success of breastfeeding practices amongst the participants.” (Sim et al., 2014, p216)  *“…because this [fenugreek] works so quickly and it just gave me that confidence straight away. It took away that anxiety and stress” (BW 6)”* (Sim et al., 2014, p216)  “Raspberry leaf tea can give women this sense of being supported, nurtured and replenished. As one woman said: *I don’t notice a huge difference as far as whether or not I have more milk or less milk, or anything like that. I think the one thing the raspberry leaf tea does is it helps me relax. Just because it’s a warm drink, I think. So I just sit down and I feel like, okay, I’ll just put my feet up, have a cup of tea. It helps me relax, and feel warm and tingly inside”* (Westfall 2003b, p 26). |
| Increases my ability for self-care | Herbal medicines (ingested) | - Canada (Westfall 2003b) | “Though the women who contributed to this study typically felt unable to evaluate the herbs' efficacy, they considered them to be of some value, for they supplied nutrients and (in the case of herbal teas) water, as well as promoting a sense of relaxation and self-efficacy.” (Westfall, 2003b, p25) |
| Restoration of mind-body balance | Herbal medicines (external applications) | - Thailand (Elter et al., 2016) | “The herbs in hot bath, such as leaves of *Nat*, release aromatic oils, which are believed to relieve mind–heart, emotional, and psychological stress. LD said ‘*the water for a hot bath is boiled with leaves of an herb named Nat. The leaves will prevent her from feeling dizzy or being intoxicated.’* Leaves of *Nat* … can be used for treating fatigue, exhaustion, psychological and emotional imbalances, and postpartum depression [and also] to ward off a malevolent spirit and to make holy water. The women in this study used both the medicinal and supernatural properties of Nat leaves to treat the mind–heart essence” (Elter et al., 2016, p253). |
| ***Women’s use of CMPs during breastfeeding – perceived benefits involving spiritual protection*** | | | |
| Spiritual protection in the postpartum period | Herbal medicines (external applications) | - Thailand (Elter et al., 2016) | “In Thailand, *Nat* leaves are also used to ward off a malevolent spirit and to make holy water. The women in this study used both the medicinal and supernatural properties of Nat leaves to treat the mind–heart essence” (Elter et al., 2016, p253) |
| ***Women’s use of CMPs during breastfeeding – perceived cultural benefits*** | | | |
| Cultural cleansing after childbirth | Herbal medicines (external applications) | - Ghana (Aborigo et al. 2012) | “Also first-time mothers are expected to go through a cultural cleansing known as *sooru* in Kasem and *kosoto* in Nankani, regardless of the bitterness of their breastmilk. The process involves the pouring of warm herbal water over the mother for a period of three days if the child is a male and for four days if the child is female” (Aborigo et al. 2012, p76) |

| 1. ***Additional themes relating to perceived benefits of women’s use of CMPs throughout the childbearing continuum*** | | | |
| --- | --- | --- | --- |
| ***Perceptions of safety regarding CMP use in pregnancy and lactation*** | | | |
| Complementary medicines are safer than pharmaceutical medications | Herbal medicines (ingested) | - Australia (Sim et al., 2014) - UK (Holst et al., 2009) - Canada (Westfall, 2003a) - Indonesia (Bali) (Wulandari & Whelan, 2011) | *“I really didn’t want to take anything harsh that could affect my baby’s health, so I was more cautious over what I take. I would say that because I was breastfeeding, I was more cautious over what I take and what I eat, and I think that using natural herbs would be safer than using chemicals”. (BW 18).* (Sim et al., 2014, p216)  *“I think if you can avoid taking chemical pharmaceutical drugs, then I would exhaust every single herbal option before I go near any pharmaceutical… because the herbal options are usually much safer, they usually don’t cause any issues in the baby. For me, in any situation I would exhaust any homeopathic or herbal remedy before I went to pharmaceuticals”. (BW 11).* (Sim et al., 2014, p216)  *“To me, it [herbal galactagogue] seems a lot safer, because when I was on Motilium®, the way how that works changes dopamine levels in the brain which then increases milk supply and prolactin. That to me has always felt like ‘Frankenstein’ sort of thing. It [fenugreek] just seems more natural. I am not concerned about any transfer to my milk, because it’s natural and it has been used for hundreds or thousands of years, really, I think over time it would have been tested and proven”. (BW 7).* (Sim et al., 2014, p216)  *‘‘It is just that herbal remedies have been around for sort of much, much longer. They have been used for thousands of years and you kind of feel that they must be safer, they haven’t been tampered within the same way as medicines’’ (P2)* (Holst et al, 2009, p227)  *‘‘Having been pregnant, having children you suddenly become a hell of a lot more careful what you wanna put in your body’’ (P6) and ‘‘I am definitely going to try anything before I start taking the drugs’’ (P3).* (Holst et al, 2009, p227)  “Many women expressed caution with regards to using any medication while pregnant. That being said, they all believed herbs to be safer than pharmaceutical drugs, as a general rule. There was a general perception that herbal medicines involved lower doses than did pharmaceutical drugs, and were also milder, simpler, more natural and had fewer side-effects” (Westfall, 2003a, p36)  *“I believe in both traditional as well as modern medicine. What I‘ve known is that modern medicine sometimes bring about side-effects, but traditional herbal doesn’t. It is because traditional herbal contains bahan alami [natural ingredients] … My mother, my grandmother, my great grandmother had used this herbal to make us healthier. And it works, without any side-effects.”* (Wulandari & Whelan, 2011, p869) |
|  | Vitamins, minerals, homoeopathic remedies and herbal supplements (CAM) available over the counter | - UK (Warriner et al., 2014) | *“There should be more information, because I don't take paracetamol and things like that; I try not to take over-the-counter medicines, so I think that there should be more information about complementary medicines for people who want to take it. Because a lot of the time I think it's not going to do any harm if you do take it because it's all natural, but when you're pregnant you just fear everything don't you?(P4)”* (Warriner et al., 2014, p141) |
| Receiving reassurance that herbal medicines are safe during pregnancy and breastfeeding | Herbal medicines (ingested) | - UK (Holst et al., 2009) - Canada (Westfall, 2003a) - Australia (Sim et al., 2014) | “One [pregnant woman] considered herself lucky because her health care personnel, though not knowing about herbal products did not condemn it, *‘I think I have been lucky going to GP’s and midwifes who may not know much about it but ‘‘if you are into that and think it will help – yeah, it is not going to do any harm.’’ ’* (P2) (Holst et al., 2009, p228)  “Some local midwives …were recommending the book [Susun Weed’s ‘Wise Woman Herbal for the Childbearing Year’] to their clients, thereby increasing the likelihood that the study participants would have been exposed to it. The midwives’ recommendations likely validated the information in the book for their clients, who trusted her opinion.” (Westfall, 2003a, pp33-34)  In the context of this discussion, health care providers included doctors and specialists, midwives, child health nurses, lactation consultants, naturopaths, as well as community pharmacists … A need for reassurance from health care providers emerged as an underlying theme as some participants elucidated their experiences and relationships with their trusted health care providers... *‘I am certainly not opposed to the idea of using herbs during breastfeeding, as long as I know and have checked with my child health nurses and doctors or even ringing up a pharmacist’ (BW 12)*” (Sim et al., 2014, p216) |
| ***Using both CMPs and concurrently accessing biomedical care promotes best care for both mother and baby*** | | | |
| Better management of maternity complications in pregnancy and birth | Herbal medicines (ingested) | - Ghana (Dako-Gyeke et al., 2013) - Thailand (Liamputtong et al., 2005) - South Africa [76] | “It was also evident that seeking non-orthodox treatment is common among conventional and non-conventional antenatal clinic attendees and it is usually sourced from pastors, spiritualists and TBA’s. […] Mostly, conventional antenatal clinic clients remained committed to biomedical care and were not likely to use any herbal therapy. Yet, few antenatal clinic attendees admit to combining herbal therapy with prescribed orthodox medication and “alternate” between them for a complementary effect that will better manage maternity complications” (Dako-Gyeke et al., 2013, p211)  “Clinic and hospital childbirth is motivated by fear of complications. *“I came to the clinic to put my name on their books, in case I have a difficult delivery.”* (Ngomane & Mulaudzi, 2012, p36)  “As women nowadays receive care under modern obstetric regimes, and have more contact with biomedicine, most women received two sources of knowledge: medical and cultural. [One woman]… said that her doctor advised her to drink lots of milk, eat food containing the ﬁve food groups and not to consume pickled food, smoke or drink alcohol. Her mother, however, told her to eat traditional food such as *pak plang* for an easy birth, and taking only half a banana so that she could avoid an obstructed birth. …  *‘I followed what other people told me to do because I wanted to make sure that the baby would be ﬁne. I was afraid that the baby would be born abnormal. If I didn’t follow their advice, and if something was wrong with the baby, it would be difﬁcult for me as a mother and it would be difﬁcult for the crippled baby’*.” (Liamputtong et al., 2005, pp148-149). |
| Protection of the baby from diseases understood to arise from spiritual causes as well as from diseases treatable with biomedical medicines | Herbal medicines (ingested) | - Swaziland (Thwala et al., 2012) - Nigeria (Okafor et al, 2014) | *“I use traditional medicines during the pregnancy . . . I also go to the hospital every month to have check-ups. They give me pills which I take home to drink together with the traditional medicines . . . When the time comes for the baby to be born . . . I . . . go to hospital to have my baby. I have never had a home birth . . . but I do assist other women in the village when they have their births at home. … [I use both traditional medicines and hospital medicines] . . . to make sure that the baby is protected on all fronts; protected from kuhabula [acquisition of illnesses from bad spirits in the environment] through the use of traditional medicine as well as protected from the hospital diseases by using their modern medicine.”* (Thwala et al., 2012, p95)  *“There is a disease, ‘Maridejo’ which attacks babies before 8 days of age, so they use the TBAs so that their babies will not be attacked by the disease because it has no cure in the hospital. They use the hospital to check their BP, weight, to know the position of the baby and other tests. Also after delivery, they take immunization in the hospital.”* (Okafor et al, 2014, p47) |
